# A network analysis of affective and motivational individual differences and error monitoring in a non-clinical sample

**DOI:** 10.1093/cercor/bhae397

**Published:** 2024-10-25

**Authors:** Anna Grabowska, Filip Sondej, Magdalena Senderecka

**Affiliations:** Doctoral School in the Social Sciences, Jagiellonian University, Main Square 34, 31-110 Krakow, Poland; Centre for Cognitive Science, Jagiellonian University, Ingardena 3, 30-060 Krakow, Poland; Centre for Cognitive Science, Jagiellonian University, Ingardena 3, 30-060 Krakow, Poland; Centre for Cognitive Science, Jagiellonian University, Ingardena 3, 30-060 Krakow, Poland

**Keywords:** error monitoring, Gaussian graphical modelling, affective traits, motivation

## Abstract

Error monitoring, which plays a crucial role in shaping adaptive behavior, is influenced by a complex interplay of affective and motivational factors. Understanding these associations often proves challenging due to the intricate nature of these variables. With the aim of addressing previous inconsistencies and methodological gaps, in this study, we utilized network analysis to investigate the relationship between affective and motivational individual differences and error monitoring. We employed six Gaussian Graphical Models on a non-clinical population ($N$ = 236) to examine the conditional dependence between the amplitude of response-related potentials (error-related negativity; correct-related negativity) and 29 self-report measures related to anxiety, depression, obsessive thoughts, compulsive behavior, and motivation while adjusting for covariates: age, handedness, and latency of error-related negativity and correct-related negativity. We then validated our results on an independent sample of 107 participants. Our findings revealed unique associations between error-related negativity amplitudes and specific traits. Notably, more pronounced error-related negativity amplitudes were associated with increased rumination and obsessing, and decreased reward sensitivity. Importantly, in our non-clinical sample, error-related negativity was not directly associated with trait anxiety. These results underscore the nuanced effects of affective and motivational traits on error processing in healthy population.

## Introduction

The process of error monitoring plays a crucial role in developing adaptive behavior, which allows our actions to be shaped by their outcomes (Ullsperger et al. 2014). People vary greatly in terms of how they interpret and appraise errors. Observation of these discrepancies is one of the cornerstones of research on individual differences in cognitive control (Meyer and Hajcak 2019). Despite strong evidence that error monitoring is modulated by a large number of individual affective and motivational traits, previous studies have typically focused on single emotional characteristics or a narrow set of them (see Moser et al. 2013; Weinberg et al. 2015, for a review). This approach, although fruitful in many cases, does not take into account the fact that affective and motivational traits are interconnected and their influence on an individual’s ability to monitor and respond to errors may be either direct or indirect. This is especially true of anxiety-related traits since “anxiety” can be considered an umbrella term that subsumes various mutually connected phenomena (Barlow 2002). Consequently, knowledge of the complex associations occurring at the system level between error monitoring and the different affect- and motivation-related concepts is still limited and requires further investigation. In the present study, we aim to tackle this challenge by applying a network analysis technique that has recently opened new exciting avenues in research on psychopathologies (Borsboom and Cramer 2013; Fried and Cramer 2017; [Bibr ref35][Bibr ref35]; Robinaugh et al. 2020) and personality (Cramer et al. 2012; Costantini et al. 2019). Our goal is to obtain a comprehensive data-driven view on the complex interplay between affect- and motivation-related individual traits and error monitoring.

### Error monitoring

A well-established biomarker of error monitoring is error-related negativity (ERN; Gehring et al. 1993, also called error negativity, Ne; Falkenstein et al. 1991). ERN is an event-related potential (ERP) component that is extracted from the electroencephalographic (EEG) signal and represents a neural response to error commission. It manifests as a sharp negative wave that occurs over mediofrontal scalp regions up to 100 ms after an erroneous motor response. ERN has traditionally been related to activity in the medial frontal cortex, specifically in the dorsal anterior cingulate cortex (dACC; Dehaene et al. 1994; Brazdil et al. 2005; Debener et al. 2005). More recent studies have indicated that ERN can also originate from the supplementary motor area (SMA; Bonini et al. 2014) or the pre-supplementary motor area (pre-SMA; Iannaccone et al. 2014; Fu et al. 2019).

There are two main groups of theories that try to explain what ERN actually is. Theories from the first category are primarily focused on cognitive aspects; they implicate ERN as a reflection of the mechanism that tracks the mismatch between an intended action and the actually executed response and leads to error detection (Gehring et al. 1993; Falkenstein et al. 2000), or as an index of conflict between simultaneously active correct and incorrect response representations (Carter et al. 1998), or as a signal of the increase in attentional control that is typically observed in situations requiring continuous monitoring of performance (Noordt et al. 2015). In turn, theories from the second category are focused on affective and motivational factors; they interpret ERN as a signal associated with negative reinforcement learning (Holroyd and Coles 2002) or rapid mobilization of defensive systems (Weinberg et al. 2012b). Theories in this category generally propose that the monitoring system responds to actions that deviate from motivational objectives or elicit a negative emotional state due to their ineffectiveness (e.g. Luu et al. 2004). This negatively valenced response can be augmented by contextual factors.

Importantly, numerous studies have indeed indicated that an individual’s affective and motivational states can influence ERN amplitude. It has been observed that ERN decreases with mental fatigue (Boksem et al. 2006). It has also been observed that ERN increases when incorrect responses are evaluated (Hajcak et al. 2005) or punished (Riesel et al. 2012), or when they are personally meaningful (Legault and Inzlicht 2013) or costly (Hajcak et al. 2005). More pronounced ERN has also been reported when measured in the context of viewing stimuli that induce short-duration affective states (e.g. Larson et al. 2006; Wiswede et al. 2009; Wouwe et al. 2011 but see Moser et al. 2005; Senderecka 2016; Paul et al. 2017; Senderecka 2018; Senderecka et al. 2018a for the null effect obtained in similarly designed studies). This implies that the performance monitoring system might have a broader role than just identifying behavioral errors or resolving conflicting response choices. Rather, it detects and evaluates errors and conflicts within the context of individual motivational goals and affective states.

It is worth noting that ERN was initially considered to be specific to errors (e.g. Gehring et al. 1993). However, later studies revealed that correct responses evoke a similar negative component as errors; this component is called correct-related negativity (CRN; Ford 1999; Vidal et al. 2000). CRN emerges shortly after the execution of a correct response and reaches its highest amplitude at the same mediofrontal electrode sites as ERN (Ford 1999; Falkenstein et al. 2000; Vidal et al. 2000, 2003). CRN has been interpreted as an index of certainty regarding the accuracy of the response made, with more-pronounced amplitudes indicating decreased certainty (Scheffers and Coles 2000; Pailing and Segalowitz 2004). It has also been identified as a reflection of elevated tendency to check response correctness (Falkenstein et al. 2000; Vidal et al. 2000), or as response-triggered engagement of cognitive control (Bartholow et al. 2005). Although ERN and CRN share some similarities, it is far from certain that they are the same component which varies in amplitude with response correctness (Vidal et al. 2021). Since ERN may reflect activity related both to general response monitoring and specific error detection, CRN should also be taken into account when examining error-related brain activity.

### ERN and individual differences: a dimensional approach

People differ widely in their ability to monitor errors and in their sensitivity to error-related affective and motivational factors. The observed variability in error-related brain activity has been linked to multiple forms of psychopathology and individual differences (for a review, see Moser et al. 2013; Weinberg et al. 2015; Meyer and Hajcak 2019). Reduced ERN amplitude has been reported in a wide range of externalizing disorders, e.g. attention-deficit hyperactivity disorder (Meel et al. 2007; Senderecka et al. 2012) and mental conditions associated with blunted affect and lack of motivation, e.g. schizophrenia (Mathalon et al. 2002; Foti et al. 2012), or characterized by impulsive response style, e.g. borderline personality disorder (Bruijn et al. 2006; Ruchsow et al. 2006). A similar relationship has been found in non-clinical populations in which increased levels of impulsivity or impulsivity-related traits such as decisiveness are accompanied by decreased ERN amplitude (Ruchsow et al. 2005; Stahl and Gibbons 2007; Senderecka et al. 2018b).

In contrast, increased ERN amplitudes have been reported in mental conditions associated with anxiety, overestimation of threat and pathological uncertainty, e.g. general anxiety disorder and obsessive-compulsive disorder (OCD; see Olvet and Hajcak 2008; Endrass and Ullsperger 2014; Riesel 2019, for a review). However, more pronounced ERN has not been consistently found in all types of anxiety and mental conditions. A meta-analysis conducted by Moser et al. (2013) showed that ERN is associated with worry-related facets of anxiety, i.e. anxious apprehension ($r = -$.35), but not with somatic anxiety, i.e. anxious arousal ($r = -$.09). Similarly, a recent meta-analysis by Saunders and Inzlicht (2020) demonstrated that the relationship between anxiety and ERN remains significant after multiple corrections for publication bias, but only for a few specific sub-types and populations of anxiety. This possibly explains why individuals with clinical phobias or post-traumatic stress disorder do not show altered ERN compared to controls (see Weinberg et al. 2015, for a review). These individuals’ traumatic experiences or persistent fear of specific objects, situations, or activities may account for sympathetic symptoms but do not translate into increased concerns over mistakes.

In turn, individuals with clinical depression, characterized by high severity of anxiety symptoms, usually show altered ERN amplitudes compared to controls. However, the direction of the association between depression and ERN amplitude is tentative, with studies reporting more pronounced (Chiu and Deldin 2007; Holmes and Pizzagalli 2008, 2010) but also blunted (Ladouceur et al. 2012; Schoenberg 2014) or unchanged ERN amplitudes (Schrijvers et al. 2008; Olvet et al. 2010; Weinberg et al. 2012a).

Anxiety-related changes in ERN amplitude are not unique to psychopathology. In non-clinical populations, individuals who are more prone to anxiety and worry tend to exhibit more-pronounced ERN amplitudes (Weinberg et al. 2012b; Moser et al. 2013; Banica et al. 2020). Riesel et al. (2017) found that participants with health anxiety showed enhanced ERN amplitudes compared to healthy controls and did not differ from OCD patients in ERN. Recently, Härpfer et al. (2022) demonstrated an association between ERN and the trait-like tendency to worry in a general population that included both clinical as well as non-clinical individuals. This suggests that an individual’s susceptibility to anxiety, even in the absence of clinical anxiety disorders, can influence error monitoring processes.

These findings suggest that increased ERN amplitudes are not specific to any particular psychopathology but are observed across various mental conditions. Consequently, ERN has been proposed as a promising transdiagnostic endophenotype for the development and maintenance of psychopathological symptoms (e.g. Riesel et al. 2019). However, this assumption has been challenged in recent studies (e.g. Härpfer et al. 2020; Muir et al. 2020; Seow et al. 2020). Chen and Itier (2024) reported no association between ERN amplitude and anxious traits in individuals with subclinical or clinical anxiety without a formal diagnosis. Similarly, the multiverse analysis of the ERN-anxiety relationship by Clayson (2024) indicates that this relationship in a general population is fragile. These results suggest that the relationship between anxiety and ERN might be stronger in clinical samples, which aligns with the findings of the meta-analysis by Saunders and Inzlicht (2020).

Taken together, the results of studies on the relationship between error-monitoring and various individual traits, in both clinical and non-clinical populations, do not present a consistent pattern of associations between ERN and anxiety-related differences (for a review, see Weinberg et al. 2015; Riesel et al. 2023; see also Pasion and Barbosa 2019; Zambrano-Vazquez et al. 2019; Macedo et al. 2021). A substantial body of evidence indicates that symptoms related to some kinds of anxiety are associated with increased ERN, and symptoms related to motivational disengagement are associated with blunted ERN. However, it is not clear exactly what kind of anxiety is associated with ERN. A variety of traits and symptoms have been discussed as being primarily associated with ERN amplitude. Among these, some of the most promising include behavioral inhibition/avoidance (Moser et al. 2013), threat/error sensitivity (Weinberg et al. 2015), harm avoidance (Riesel et al. 2019), uncertainty (Cavanagh and Shackman 2015; Ruchensky et al. 2020), impulsiveness (Taylor et al. 2018; Overmeyer et al. 2021), rumination (Tanovic et al. 2017), and checking symptoms (Weinberg et al. 2015). Developmental research suggests that ERN amplitude is associated with error sensitivity, which is presumably linked to parenting style and stressful childhood experiences (e.g. Chong and Meyer 2019; Chong et al. 2020; Mehra et al. 2022). As already mentioned, some studies have related ERN variability to anxious apprehension (e.g. Moser et al. 2013). Nevertheless, there is strong evidence that anxious apprehension is a complex phenomenon involving narrower symptoms, such as worry, negative affect, or sensitivity to threat (Olatunji et al. 2010; Sharp et al. 2015), and ERN may not be uniformly linked to all of them.

In an attempt to understand these contradictory results regarding ERN, it has recently been emphasized that ERN may re-affect the end result of a complex process that integrates information about threat, reward, punishment, and the need for cognitive control (Weinberg et al. 2015). In most cases, these various motivational and cognitive variables co-occur, making it difficult to determine direct relationships between these variables and changes in ERN amplitude. Attempts at a dimensional approach to anxiety-related psychopathologies, individual differences, and ERN have already been made, but without conclusive results. In 2016, Weinberg and colleagues proposed a model of the evaluative and compensatory components of the error monitoring process that defines ERN as a biomarker of the early evaluative stage in executive control instead of as a reflection of the degree of instantiated cognitive control (Weinberg et al. 2016). This model links the magnitude of this evaluative response to sensitivity to both contextual factors and individual differences and suggests that the ERN amplitude may reflect an individual’s sensitivity to endogenous threat (Weinberg et al. 2012b, 2016). Following this, on the basis of an extensive study involving non-clinical adolescents, Weinberg et al. (2016) revealed that *checking* behaviors constitute a specific dimension of anxiety that may, to some extent, account for the more pronounced ERN amplitudes observed in anxious individuals. Recently, in an extensive study involving participants with a spectrum of obsessive–compulsive characteristics and data from twenty-five self-report questionnaires, Riesel et al. (2023) demonstrated a specific association between ERN and OC symptom severity, as well as with the anxious-misery latent factor. In turn, [Bibr ref177][Bibr ref177][Bibr ref177] examined the relationship between ERN amplitude and nine anxiety-related constructs within a non-clinical population, but they were unable to replicate previous findings regarding the association between ERN and anxiety. Thus, the question about the specific relationship between error monitoring and the different dimensions of anxiety is still open.

### The current study

Following this dimensional line of work, the first aim of the present study was to identify the specific symptoms that are associated with altered ERN amplitude. Using the network analysis approach (e.g. Borsboom and Cramer 2013; Huster et al. 2020), with 260 healthy individuals constituting the training set, we examined associations between ERN and self-report measures related to anxiety, depression, obsessive thoughts, compulsive behavior, motivation, reward, and punishment; we then validated our results on an independent set of 112 healthy participants from the general population.

The network approach allows graph estimation in which conditional dependencies between variables (e.g. neurophysiological, cognitive, affective, social, etc.) are depicted in a network of nodes. The presence of an edge connecting any two nodes signifies the presence of a distinct and direct relation that remains intact even when potential shared dependencies on other nodes in the network are taken into account. Importantly, network analysis makes it possible to analyze the associations between ERN and variables related to reward, punishment, or anxiety simultaneously. Furthermore, the number and strength of the connections among individual variables can offer insights into their overall significance within the network, enabling the identification of variables that have a strong influence on error monitoring (Opsahl et al. 2010; Bringmann and Eronen 2018).

In an attempt to refine the individual differences-ERN association, we first examined the network of affect- and motivation-related traits, assessed on the basis of both self-report measures and ERN. Following this, we examined the network of affect- and motivation-related traits and CRN and determined relationships that are unique to the ERN network in order to separate the relationships associated with error-related brain activity from those associated with response-related brain activity. Drawing on the advantages of the network approach, the second aim of the present study was to examine the structure of the estimated ERN networks to better understand the interplay of variables related to anxiety, depression, reward, and punishment.

## Materials and methods

### Participants

The present study involved analyses of datasets that were gradually collected at different time points within a larger project conducted in our laboratory. Since power analysis is utilized to determine the necessary sample size required to detect a statistically significant effect and is typically not employed in the context of cross-validation, we did not determine the sample size a priori. Instead, we assessed the performance and generalizability of our models by splitting the available data into training and testing subsets. Our initial dataset consisted of 399 volunteers (236 females, 162 males, 1 non-binary gender) aged 18–40 (*M* = 23.26, SD = 3.95). Part of this dataset ($N$ = 171) was utilized in a previous analysis, described in Grabowska et al. (2024a), whereas a separate part ($N$ = 225) was used in a feedback-related study described in Grabowska et al. (2024b).

Participants were recruited from the general population via internet advertisements. All participants were healthy, free of medications, declared no history of neurological or psychiatric diseases, and had normal or corrected-to-normal vision. The average number of years of education of the participants was 15.41 (SD = 2.40). Prior to analysis, 27 participants were excluded for the following reasons: lack of EEG data registration owing to technical problems ($N$ = 3); no responses during the task ($N$ = 1); less than five committed errors ($N$ = 10); low overall EEG data quality ($N$ = 4); less than five trials that contained an erroneous response after the artifact rejection procedure ($N$ = 9); internal consistency of error-related brain signal lower than.70 ($N$ = 29). The final sample consisted of EEG data from 343 participants (198 females, 144 males, 1 non-binary gender) aged 18–40 (*M* = 23.34, SD = 4.01). The average number of years of education among the participants in the final sample was 15.34 (SD = 2.51). The final sample was then divided into a training set ($N$ = 236) and a testing set ($N$ = 107) to perform network stability tests.

### Procedure and task

Participants received verbal and written information about the purpose and procedure of the study. The study was performed following the Declaration of Helsinki (BMJ 1996); the protocol was approved by the Research Ethics Committee at the Philosophical Faculty of Jagiellonian University in Kraków, Poland. All participants provided written informed consent and were monetarily compensated for their time.

**Fig. 1:**
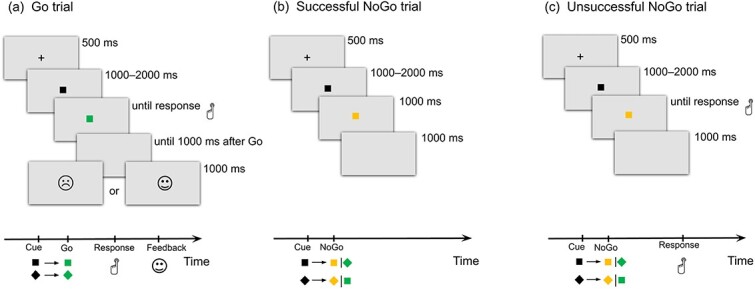
Scheme of the go/no-go task used and its conditions: go trial (a); successful no-go trial (b); unsuccessful no-go trial, i.e. with erroneous response (c).

While the EEG signal was recorded, participants performed a speeded color plus orientation discrimination task that has previously been validated in several studies (Vocat et al. 2008; Pourtois 2011). They had to press the response key as quickly as possible with their right thumb if the black geometric figure (square or diamond) turned green and kept the same spatial orientation (two-thirds of the trials, all corresponding to go trials). By contrast, they were asked to withhold their response if the black figure turned green but changed orientation, or if it turned orange, irrespective of its orientation (one-third of the trials, all corresponding to no-go trials). The task included a training block (15 trials), four experimental sessions of 84 trials each, and two calibration blocks of 14 trials, which preceded the first and third experimental blocks. During each calibration block, the mean RT for go trials was calculated online and used to define a threshold for positive and negative feedback (a smiling or sad face). To receive positive feedback during the first two experimental blocks, participants had to be 10% faster than the mean RT calculated during the first calibration block; during the third and fourth experimental blocks, participants had to be, respectively, 10 or 20% faster than the mean RT calculated during the second calibration block. Participants received feedback about the speed of their responses only on go trials. This feedback was intentionally unrelated to performance correctness, as Olvet and Hajcak (2009a) found that correctness-related feedback could influence the relationship between ERN amplitude and anxiety. In no-go trials, where participants were expected to make commission errors, feedback was not provided to enhance internal performance monitoring. Frequent speed-related feedback was used to maintain a reasonable level of error rate across participants, consistent with the recommendations of Gehring et al. (2012). It is also worth noting that speed-related trial-to-trial feedback has been frequently used in previous ERN-related studies, including those investigating internalizing symptoms (Endrass et al. 2008, 2010; Aarts et al. 2013). All stimuli were displayed in the center of a gray screen for the time intervals shown in Fig. 1. The instructions and the entire experiment were in the participants’ native language.

### Questionnaires

From the set of questionnaires (Polish adaptations or Polish translations made using a forward-backward translation protocol) completed by participants, the following were selected for analyses of anxiety-related phenomena:

Rumination-Reflection Questionnaire (RRQ; Trapnell and Campbell 1999), rumination subscale; State-Trait Anxiety Inventory (STAI; Spielberger et al. 1970, 2006), trait and state subscales (STAI state was measured twice, once before the go/no-go task was performed, and the second time immediately after; the difference between these two measurements was used to create an affective load variable); Depression Anxiety Stress Scales—21 (DASS-21; Lovibond and Lovibond 1995; Antony et al. 1998), depression and stress subscales; Behavioral Inhibition System Scale (Carver and White 1994; Müller and Wytykowska 2005, BIS;); Behavioral Activation System Scale (BAS; Carver and White 1994; Müller and Wytykowska 2005), drive, reward responsiveness, and fun-seeking subscales; Obsessive-Compulsive Inventory-Revised (OCI-R; Foa et al. 2002; Huppert et al. 2007), checking, obsessing, ordering, neutralizing, and washing subscales; Obsessive Beliefs Questionnaire—20 (OBQ-20; Moulding et al. 2011), inflated responsibility for harm, importance/control of thoughts, overestimation of the threat, and perfectionism/intolerance of uncertainty subscales; White Bear Suppression Inventory (WBSI; Wegner and Zanakos 1994; Cichoń et al. 2020); Intolerance of Uncertainty Scale—12 (IUS-12; Carleton et al. 2007), prospective and inhibitory intolerance of uncertainty subscales; Indecisiveness Scale (IND; Frost and Shows 1993); Sensitivity to Punishment and Sensitivity to Reward Questionnaire (SPSRQ; Torrubia et al. 2001), punishment and reward sensitivity subscales; Frost Multidimensional Perfectionism Scale (FMPS; Frost et al. 1990), personal standards subscale; Guilt Sensitivity Questionnaire (GSQ; Melli et al. 2017); Need for Closure Scale (NFCS; Webster and Kruglanski 1994; Kossowska 2003), avoidance of ambiguity and need for predictability subscales; Maximization Scale (MS; Schwartz et al. 2002), high standards subscale; Rosenberg Self-Esteem Scale (SES; Rosenberg 1989; Laguna et al. 2007).

All scoring was performed in line with instructions provided by the authors of the questionnaires.

### Electrophysiological recording and data pre-processing

The experiment was conducted by trained researchers in a sound-attenuated room. The EEG signal was continuously recorded at 256 Hz from 64 silver/silver-chloride (Ag/AgCl) active electrodes (with preamplifiers) using the BioSemi Active-Two system and referenced online to the Common Mode Sense–Driven Right Leg ground, which drives the average potential across all electrodes as closely as possible to amplifier zero. The horizontal and vertical electro-oculograms were monitored using four additional electrodes placed above and below the left eye and in the external canthi of both eyes after adequate skin preparation. The EEG signal was pre-processed with BrainVision Software (Brain Products GmbH, Gilching, Germany 2021). The signal was re-referenced off-line to the average of the left and right mastoid electrodes; it was initially filtered with a Butterworth fourth-order filter with a high pass of 0.05 Hz, and a Butterworth second-order filter with a low-pass of 128 Hz. Power-line noise was removed with a notch filter at 50 Hz. Data were further epoched around the response onset (−100 to 600 ms) and were baseline corrected to the average pre-response activity from −100 to 0 ms. The choice of a baseline window was influenced by several previous studies in the field, including those by Dimoska et al. (2006); Cavanagh et al. (2010); Boldt and Yeung (2015); Nigbur and Ullsperger (2020), and Stahl (2010). Additionally, we considered findings from electromyography-based studies, which indicate that motor time, i.e. the interval with visible changes in electromyographic activity preceding the overt mechanical response, varies between response conditions, with typical durations of approximately 180 ms for erroneous responses and 80 ms for correct responses (Allain et al. 2004; Korolczuk et al. 2024). Therefore, a window of 100 ms before response onset ensures that the baseline activity in both response conditions is more comparable and captures the neural correlates of the motor phase of the movement. Ocular artifact correction was performed with Gratton, Coles, and Donchin’s algorithm (Gratton et al. 1983). The signal was then additionally filtered with a Butterworth sixth-order filter at 40 Hz. Noise trials and channels were identified via an automatic procedure with the AutoReject MNE package (Jas et al. 2017). Specifically, AutoReject estimates a threshold $\tau $ in $\mu $V for rejection of noisy epochs using cross-validation. Then, trials with more than $\kappa $ channels marked as bad are dropped, and the worst $\rho $ trial-wise bad channels are interpolated. The exact values of $\kappa $ and $\rho $ are not pre-selected but learned from the data. Additionally, we rejected trials if the Fz channel (the key channel in our analyses) was marked as bad but not interpolated during AutoReject’s automatic procedure.

ERN and CRN amplitudes were quantified as the mean activity from 0 to 100 ms at electrode Fz. The choice of the Fz electrode was guided by previous literature (Ridderinkhof et al. 2003; Hajcak et al. 2008; Hirsh and Inzlicht 2010; Stern et al. 2010; Di Gregorio et al. 2022) and by inspecting the topographical distribution of maximal scalp activity. Selecting the electrode where activity is maximal is a common practice in ERN research (e.g. Ridderinkhof et al. 2003; Hajcak et al. 2008; Stern et al. 2010). Time windows for the targeted ERP components were selected based on our previous study using a similar experimental paradigm that requires response inhibition (Senderecka 2016).

The ERPs’ consistency was further tested. The within-subject variability and the between-subject variability of the ERPs were calculated as the standard deviation of scores in trials within one participant and between participants, respectively. Subject-level internal consistency was calculated as a subject-level dependability coefficient (Clayson et al. 2021). Participants that had internal consistency of ERPs lower than.70 were then excluded from the analyses.

Before any further analyses, the dataset was divided into training (*N* = 236; 132 F; age: *M* = 23.28, SD = 4.06) and testing (*N* = 107; 66 F; age: *M* = 23.45, SD = 3.93) sets. The average number of artifact-free epochs consisting of an erroneous or a correct response that were included in the analysis per participant in the training set was 27.92 (SD = 14.71) and 209.62 (SD = 15.39), respectively; in the testing set, it was 26.55 (SD = 13.99) and 212.31 (SD = 13.26). Furthermore, epochs were averaged by condition to yield correct-response and erroneous-response ERPs for each participant.

### Covariates

The choice of covariates was based on a literature review. We included age (e.g. Meyer et al. 2011), handedness (Lin et al. 2015; Kumar et al. 2020), performance measured as the ratio of successfully inhibited responses to uninhibited no-go responses (Derakshan and Eysenck 2009; Fischer et al. 2017), and ERP component latency (Johannes et al. 2001; Chang et al. 2009; Speed et al. 2017) measured as 50% fractional area latency, as recommended by Luck (2014).

### Statistical analyses

The analyses were performed using the MNE-Python (Gramfort et al. 2013), Pandas (McKinney et al. 2010; Reback et al. 2021), Scikit-learn (Pedregosa et al. 2011), NumPy (Harris et al. 2020), skggm (Laska and Narayan 2017), and networkX (Hagberg et al. 2008) Python programming language packages. Skewed distributions of questionnaires, ERPs, and covariate scores were normalized using the BoxCox transformation (Box and Cox 1964). Due to the highly non-normal distributions, neutralizing and washing symptoms from the OCI-R scale were excluded from the analyses in order to meet the requirements of Gaussian Graphical Models.

#### Network estimation

To estimate the relationships between ERN and anxiety symptoms and the influence of covariates on these relationships, we created four network models consisting of the following variables: (1) ERN and anxiety symptoms; (2) ERN, anxiety symptoms, and covariates; (3) CRN and anxiety symptoms; (4) CRN, anxiety symptoms, and covariates. Some of the variability in error-related brain activity can be attributed to response giving. Thus, to extract associations that are unique to errors, we created additional two networks that contained both ERN and CRN amplitudes: (5) ERN, CRN, and anxiety symptoms; (6) ERN, CRN, anxiety symptoms, and covariates.

The structure of these networks was estimated using adaptive random Lasso regularized Gaussian Graphical Models (also called Markov Models; Lauritzen 1996) with stability selection (Meinshausen and Bühlmann 2010). In Gaussian Graphical Models, variables are considered *nodes*, and *edges* represent conditional dependence relations; these conditional dependence relations can be interpreted as partial correlation. Zero entries in the inverse covariance matrix indicate that two variables are conditionally independent, given all other variables in the model. The Lasso, that is the least absolute shrinkage and selection operator (Santosa and Symes 1986; Tibshirani 1996), can be used in inverse covariance matrix estimation to force a sparse network structure and thus minimize the number of false positive findings; this procedure is called *graphical Lasso* (Friedman et al. 2008). The strength of the sparsity is controlled by a penalty parameter $\lambda $. Network structure estimated with simple regularization is characterized by low stability, which is a natural feature of all sparse solutions. As Xu et al. (2012) pointed out, a sparse solution cannot be stable (and vice versa, a stable solution cannot be sparse). Thus, to estimate network structures with greater confidence, we used an adaptive graphical Lasso procedure (see Zhou et al. 2011; see also Zou 2006; Meinshausen 2007, which consists of three steps: (1) calculating the initial support estimate; (2) calculating the initial inverse covariance matrix with a graphical Lasso; (3) refining the initial inverse covariance matrix using the initial support estimate. The initial support estimate is computed using a form of ensemble model averaging called stability selection, combined with randomized model selection (i.e. selection of penalty parameter) using random Lasso (Meinshausen and Bühlmann 2010; Wang et al. 2011). This technique estimates *N* inverse covariance matrices created from an ensemble of *N* graphical Lasso estimators with random penalty parameters and random bootstrapped samples. Based on these *N* inverse covariance matrices, a proportion matrix is created that shows the probability of being non-zero for each edge in the network. Then, inverse covariance support, i.e. the initial support estimate, is estimated by converting the proportion matrix to binary form using the chosen probability threshold value: edges whose probability of being non-zero exceeds the threshold value are converted to 1; edges whose probability of being non-zero does not exceed the threshold value are zeroed. The inverse covariance support is further used to refit the initial network structure. In the presented work, we used 1000 bootstrapped samples and adopted a threshold of 65% for the stability selection procedure. A detailed description of the adaptive inverse covariance estimation procedure can be found in Laska and Narayan (2016).

The networks’ structures were visualized using the networkX Python package. Blue edges represent negative conditional dependence relationships between variables; red edges represent positive ones.

For each network, we assessed the nodes’ predictability, which measures how effectively a given node can be predicted by its neighbors. The predictability metric corresponds to $R^{2}$, indicating the proportion of a node’s variance that is explained by its adjacent nodes (Haslbeck and Fried 2017; Haslbeck and Waldorp 2018). We also estimated the degree centrality (Diestel 2005) and current flow closeness centrality (also known as information centrality; Stephenson and Zelen 1989) of each node to identify the most influential nodes in the whole network. Degree centrality for a node is defined as the fraction of nodes it is connected to. Current flow closeness centrality for a node is a variant of closeness centrality, based on effective resistance between nodes in a network. Rather than flowing along the shortest paths *only*, current flow closeness centrality assumes that information spreads efficiently like an electrical current. Thus, current flow closeness centrality is based on the information (i.e, the content of linkages) that is contained in all possible paths between pairs of nodes, not only information that is contained along the shortest path. For a broader discussion on current flow centrality measures, see Brandes et al. (2005).

To reduce the dimensionality of estimated networks and thus facilitate the high-level analysis of the network structures, we calculated the maximal spanning tree using Prim’s algorithm (Jarník 1930; Prim 1957).

Estimation of networks, especially ones that consist of many nodes, requires estimation of many parameters, usually using a limited sample size. In light of concerns regarding network accuracy, Epskamp et al. (2018) proposed that researchers should assess the accuracy and stability of edge weights and centrality measures in order to ensure the validity of conclusions. We followed Epskamp et al.’s proposal and expanded the networks’ accuracy estimates for an additional test. The stability of the networks’ structures was estimated via (1) a stability selection procedure that provides information on the stability of the edges in networks estimated with random penalties and random subsamples; (2) the similarity of the estimated networks to networks estimated on the testing dataset, calculated as Pearson’s *r* correlation coefficient between adjacency matrices (e.g. Fried et al. 2020); (3) an additional bootstrapping procedure performed outside the stability selection. Using 1000 bootstrapped samples, we estimated the stability of the edge weights as bootstrapped 95% CI intervals around the estimated edge weights. We then estimated the sensitivity and specificity of the edges. The stability of the selected network measures was estimated using the procedure described by Costenbader and Valente (2003) and recommended by Epskamp et al. (2018) for psychological networks.

The code to reproduce all analyses is available at https://github.com/abelowska/errorNet and at https://github.com/abelowska/skggmNets under the MIT licenses. The dataset consisting of EEG recordings and questionnaire data along with additional online resources is available at https://osf.io/nfwb6/; we also provide all model outputs, such as precision matrices, to make the analyses reproducible.

## Results

### Behavioral measures

The average number of erroneous no-go and correct go responses per participant in the training set was 32.20 (SD = 14.91) and 222.34 (SD = 3.12), respectively. In the testing set, it was 30.48 (SD = 15.02) and 222.21 (SD = 6.51) for erroneous no-go and correct go responses, respectively. In the training dataset, the average RT for incorrect no-go responses was 269 ms (SD = 40 ms), whereas for correct go responses, it was 313 ms (SD = 39 ms). As expected, participants responded significantly faster on erroneous no-go trials relative to correct go trials (*t*(235) = −29.90, $P$ <.001). In the testing dataset, the average RT for incorrect no-go responses was 279 ms (SD = 50 ms), whereas for correct go responses, it was 317 ms (SD = 39 ms). Again, participants responded significantly faster on erroneous no-go trials relative to correct go trials (*t*(106) = −14.40, $P$ <.001). The average performance score, calculated as the ratio of inhibited no-go responses to uninhibited no-go responses, was .71 (SD = 0.13) and .73 (SD = 0.13) in the training and testing sets, respectively.

Table 1 displays descriptive statistics for self-report measures, covariates, and ERP amplitudes within the training dataset. Distributions of self-report measures, covariates, and ERP amplitudes in the training and testing datasets are shown in supplementary materials.

**Table 1:** Descriptive statistics for questionnaire measures, covariates, and ERP amplitudes in the training dataset.

		M	SD	Min	Max	*P*-value	Consistency
Scales	Rumination	.78	.78	.78	.78	.78	.92
	Stress	.42	.42	.42	.42	.42	.86
	Depression	.35	.35	.35	.35	.35	.87
	Trait anxiety	.45	.45	.45	.45	.45	.88
	Affective load	.48	.48	.48	.48	.48	.91/.92
	Behavioral inhibition	.77	.77	.77	.77	.77	.79
	Obsessing	.41	.41	.41	.41	.41	.84
	Hoarding	.16	.16	.16	.16	.16	.72
	Ordering	.88	.88	.88	.88	.88	.83
	Checking	.63	.63	.63	.63	.63	.73
	Washing	.78	.78	.78	.78	.78	.69
	Neutralizing	.61	.61	.61	.61	.61	.54
	Thought supression	.48	.48	.48	.48	.48	.90
	Prospective intolerance of uncertainty	.28	.28	.28	.28	.28	.78
	Inhibitory intolerance of uncertainty	.57	.57	.57	.57	.57	.84
	Self-esteem	.66	.66	.66	.66	.66	.90
	Drive dimension of BAS	.39	.39	.39	.39	.39	.77
	Fun seeking dimension of BAS	.25	.25	.25	.25	.25	.66
	Reward responsiveness dimension of BAS	.70	.70	.70	.70	.70	.58
	Indecisivness	.92	.92	.92	.92	.92	.88
	Punishment sensitivity	.66	.66	.66	.66	.66	.83
	Reward sensitivity	.04	.04	.04	.04	.04	.64
	Inflated harm responsibility	.78	.78	.78	.78	.78	.49
	Importance of thought control	.24	.24	.24	.24	.24	.70
	Threat overestimation	.25	.25	.25	.25	.25	.74
	Perfectionism/intolerance of uncertainty	.92	.92	.92	.92	.92	.75
	Personal standards	.47	.47	.47	.47	.47	.85
	Guilt sensitivity	.31	.31	.31	.31	.31	.85
	Avoidance of ambiguity	.56	.56	.56	.56	.56	.68
	Need for predictability	.55	.55	.55	.55	.55	.79
	High standards	.96	.96	.96	.96	.96	.67
Covariates	Age	23.28	4.06	18.00	40.00	.73	–
	Handedness	0.91	0.29	0.00	1.00	.39	–
	Performance	3.73	3.25	0.26	36.33	.85	–
	ERN latency (ms)	46.51	27.85	−42.19	121.88	.19	.66 –.98 (*Med* =.92)
	CRN latency (ms)	66.10	58.96	−50.00	172.66	.06	.99 – 1.00 (*Med* =.99)
ERP amplitudes	ERN	−3.67	4.49	−15.80	13.73	.37	.70 –.97 (*Med* =.88)
	CRN	1.16	3.90	−9.99	12.28	.37	.95 –.99 (*Med* =.98)

*Note.* The questionnaires used to collect the self-report measures are described in the Materials and methods section. BAS = behavioral activation; Performance = ratio of inhibited to uninhibited no-go responses; Handedness = self-declared, 1 corresponds to right-handedness, 0 corresponds to left-handedness; ERN = error-related negativity; ERN amplitude = mean ERN amplitude at Fz (0–100 ms); ERN latency = 50% fractional area latency of ERN; CRN = correct-related negativity; CRN amplitude = mean CRN amplitude at Fz (0–100 ms); CRN latency = 50% fractional area latency of CRN; *P*-value = result of statistical test for difference between the training and testing datasets, performed with permutation tests. For interpretability purposes, all self-report measures except affective load are normalized in such a way that 0 corresponds to the smallest and 1 to the largest possible value that can be obtained. The consistency values reported for the questionnaires were measured using Cronbach’s $\alpha $. For affective load, consistencies are reported for both the first and second assessments of state anxiety, respectively. Consistencies of amplitudes and latencies are calculated using the dependability coefficient. **Table source data 1.**https://osf.io/kn4za/**Table source code 1.**https://bit.ly/3PszNtH

### ERP measures

There was a significant effect of response type (erroneous no-go vs correct go) on mean ERP amplitude at Fz in the selected time window of 0–100 ms after the response onset for the training (*t*(235) = −19.21, $P$ <.001) and testing (*t*(111) = −11.58, $P$ <.001) sets. The average ERN amplitude in the training set was −3.67 $\mu $V (SD = 4.48 $\mu $V); in the testing set it was −4.15 $\mu $V (SD = 4.92 $\mu $V). The average CRN amplitude in the training set was 1.16 $\mu $V (SD = 3.88 $\mu $V); in the testing set it was .75 $\mu $V (SD = 3.89 $\mu $V). There were no statistical differences in ERN and CRN amplitudes between the training and testing datasets (*t* = .90, *P* = .368; *t* = .90, *P* = .366 for ERN and CRN, respectively).

The within-subject variability of ERN amplitudes in the training set ranged from 3.92 to 15.51 $\mu $V (*M* = 8.25 $\mu $V; SD = 1.94 $\mu $V), and in the testing set from 4.63 $\mu $V to 14.34 $\mu $V (*M* = 8.55 $\mu $V; SD = 2.04 $\mu $V). Internal consistency of ERN amplitudes calculated as a dependability coefficient across the 236 participants in the training set ranged from .70 to .97 (*M* = .87; SD = .07); across the 107 participants in the testing set, it ranged from .70 to .98 (*M* = .87; SD = .07). The within-subject variability of CRN amplitudes in the training set ranged from 4.75 to 13.07 $\mu $V (*M* = 8.33 $\mu $V; SD = 1.53 $\mu $V) and in the testing set from 5.46 $\mu $V to 13.11 $\mu $V (*M* = 8.49 $\mu $V; SD = 1.53 $\mu $V). Internal consistency of CRN amplitudes, calculated as a dependability coefficient across the 236 participants in the training set, ranged from .95 to .99 (*M* = .98; SD = .01); across the 107 participants in the testing set, it ranged from .95 to .99 (*M* = .98; SD = .01).

**Fig. 2:**
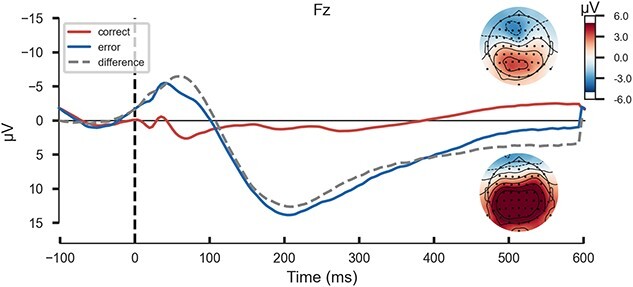
Grand average waveforms at electrode site Fz for the ERN (blue), CRN (red), and difference waveform (dashed, gray) in the training dataset ($N$ = 236). The headmaps show the topographies of response-related brain activity depicting the mean activity in the time window from 0 to 100 ms after erroneous (upper headmap) and correct (lower headmap) responses.

The grand average waveform of ERN and CRN are shown in Fig. 2.

### ERN models

**Fig. 3:**
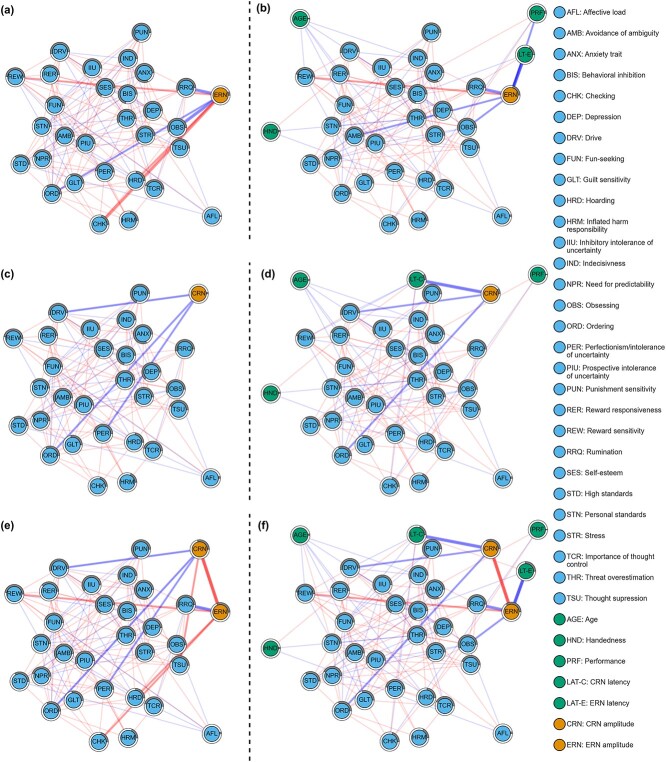
ERN network without covariates (a) and with covariates (b). CRN network without covariates (c) and with covariates (d). ERN network with control for the shared CRN variance without covariates (e) and with covariates (f). Blue edges represent negative partial correlation associations; red edges represent positive partial correlation associations. A gray shaded area around a node represents the predictability of this node, i.e. the proportion of variance in that variable explained by the network model. **Figure source data 1**. Networks: *graphs* at https://osf.io/cxkqj/**Figure source data 2**. Precision matrices: *precision_matrices* at https://osf.io/cxkqj/**Figure source code 1**. https://bit.ly/43secaj**Figure source code 2**. https://bit.ly/3vl1j5l

Unless stated otherwise, a negative association between a variable and ERN implies more negative ERN amplitude associated with an increased variable score. All references to the statistical difference test pertain to statistical tests conducted on samples generated from the bootstrapping procedure. Fig. 3 shows the relationship between ERN amplitude and selected individual differences, both with and without covariates (a), i.e. age, handedness, ERN latency, and performance (b). In the network without covariates (Fig. 3a), the ERN amplitude was directly connected with six self-report measures: it was negatively associated with rumination, obsessing, and ordering; it was positively associated with reward sensitivity, checking, and hoarding. All questionnaire variables were highly related to each other; the highest partial correlations were between trait anxiety and self-esteem (−.41), and between stress and depression (.42). Behavioral inhibition and ordering had the highest degree centrality scores (.55 and .48, respectively). Given that degree centrality reflects the fraction of node’s connections, the highest degree centrality of these two nodes suggests that they were the most connected nodes within the network. In turn, trait anxiety yielded the highest predictability scores on both the training and testing sets, at 78.83 and 81.20%, respectively. These scores indicate that the constructed network accounts for approximately 80% of the variability observed in the trait anxiety measure. Furthermore, this result demonstrates stability across different datasets. The predictability of the mean ERN amplitude was 6.28% in the training set and 6.28 % in the testing set.

When correcting for covariates (Fig. 3b), the new relationship emerged, between ERN amplitude and avoidance of ambiguity. However, the bootstrapping edge-weight difference test revealed that this change was not significant (*P* = .087). The direct relationships between ERN amplitude and ordering, hoarding, and checking disappeared ($P$ <.05, *P* = .012, $P$ <.001, respectively). From the covariates, ERN amplitude shared an edge with performance (partial correlation coefficient of −.02) and a strong edge only with ERN latency (partial correlation coefficient of −.5). Besides mean ERN amplitude, ERN latency was directly associated with behavioral inhibition and prospective intolerance of uncertainty. In turn, performance was associated with high standards (.03), hoarding (.03), and checking (−.03). Age was associated with obsessing, hoarding, fun-seeking and reward responsiveness facets of behavioral activation, indecisiveness, and reward sensitivity, with the highest partial correlations with fun-seeking and reward responsiveness (−.10). Handedness was associated with affective load, the reward responsiveness facet of behavioral activation, and threat overestimation, but most of these relationships were weak (−.02, .04, −.02, respectively). Again, behavioral inhibition had the highest degree centrality score (.48), but prospective intolerance of uncertainty had the second highest score (.39); the centrality of ordering dropped from .45 to .30 ($P$ <.001). Trait anxiety still had the highest predictability score (78.58 and 81.20% for the training and testing sets, respectively). After covariate correction, the hold-out predictability of ERN amplitude increased from 6.28% to 20.66%. This increase suggests that ERN latency shares more than 12% of the variance with ERN amplitude.

### CRN models

Fig. 3 shows the relationship between mean CRN amplitude and the scores of the selected questionnaires, without covariates (c) and with covariates (d). In the network without covariates (Fig. 3c), mean CRN amplitude showed a negative association with ordering, the drive dimension of behavioral activation and perfectionism/intolerance of uncertainty. Again, all questionnaire variables were highly related to each other, with the highest partial correlation between trait anxiety and self-esteem (−.43). The second highest partial correlation was between stress and depression (.40). The behavioral inhibition and drive dimension of behavioral activation had the highest degree centrality scores (.48). In the CRN network, trait anxiety continued to have the highest predictability score (78.83 and 81.20% for training and testing sets, respectively). The predictability of mean CRN amplitude was 3.28% in the training set, and 2.39% in the testing set.

After accounting for covariates (Fig. 3d), the association between CRN amplitude and perfectionism/intolerance of uncertainty disappeared. The bootstrapped difference test for edge-weights revealed that this change was significant ($P$ <.001). The association between CRN amplitude and ordering and drive remains the same. In terms of the covariates, CRN amplitude shared a strong edge only with CRN latency (partial correlation coefficient of −.37). CRN latency shared negative edges with the drive facet of behavioral activation, checking, and reward sensitivity scores, and positive edges with the reward responsiveness facet of behavioral activation and perfectionism/intolerance of uncertainty. Performance was negatively associated with checking (−.03) scores and positively associated with personal standards (.03) and hoarding (.03) scores. The associations between performance and other variables were consistent between the ERN and CRN models. Age and handedness had the same pattern of associations as in the ERN model. Similarly to the CRN model without covariates (Fig. 3c), the behavioral inhibition and prospective intolerance of uncertainty had the highest degree centrality scores, but they were lower (.45 and .36, respectively). The results of the bootstrapped difference test for centrality suggest that this attenuation is significant (both $P$ < .001). The predictability of trait anxiety was the same as in the ERN model with covariates (*P* = .228). These findings imply that both the ERN and CRN networks similarly explain the variability in trait anxiety. After covariate correction, the hold-out predictability of CRN amplitude increased from 2.39% to 8.86%.

### ERN and CRN models

Some of the relationships between ERN/CRN and individual differences were shared by these two ERP components. To better understand the associations between ERN, CRN and individual differences, while accounting for the shared variance between ERN and CRN, we created networks that included both ERN and CRN amplitudes as nodes. Fig. 3 shows the associations between mean ERN amplitude, mean CRN amplitude, and selected questionnaire scores without covariates (Fig. 3e) and with covariates (Fig. 3f). In the ERN/CRN network without covariates (Fig. 3e), more pronounced ERN amplitudes were specifically associated with higher levels of rumination (−.10) and lower levels of hoarding (.02), checking (.3), and reward sensitivity (.03). Thus, when controlling for CRN variance, the associations between ERN amplitudes and ordering and obsessing disappeared (all $P$ <.001). More pronounced CRN amplitudes were associated with higher levels of ordering, the drive facet of behavioral activation, and perfectionism/intolerance of uncertainty, as well as lower levels of thought suppression. Controlling for ERN variance revealed a previously unobserved relationship between CRN amplitudes and thought suppression (*P* = .002). Behavioral inhibition and the drive facet of behavioral activation had the highest degree centrality measures (.47); trait anxiety continued to have highest out-of-sample predictability score (81.20%).

After adjusting for covariates (Fig. 3f), more pronounced ERN amplitudes were associated with higher levels of rumination, longer ERN latencies, and lower levels of obsessing and reward sensitivity. The relationships between ERN amplitude and both hoarding and checking disappeared, similar to the effects observed when adjusting the ERN network for covariates (Fig. 3b). Comparing the network to the ERN with covariates network (Fig. 3b), the overall pattern of results remains similar; controlling for CRN removed only the association between ERN amplitudes and the avoidance of ambiguity node (but this difference was not significant; *P* = .368). In turn, more pronounced CRN amplitudes were associated with higher levels of ordering, the drive facet of behavioral activation, and longer latencies; these results were also observed without adjusting for ERN amplitudes. Behavioral inhibition had the highest degree centrality measures (.46), but the second highest degree centrality had the fun-seeking dimension of the behavioral activation node (.37).

### Network analysis

**Fig. 4:**
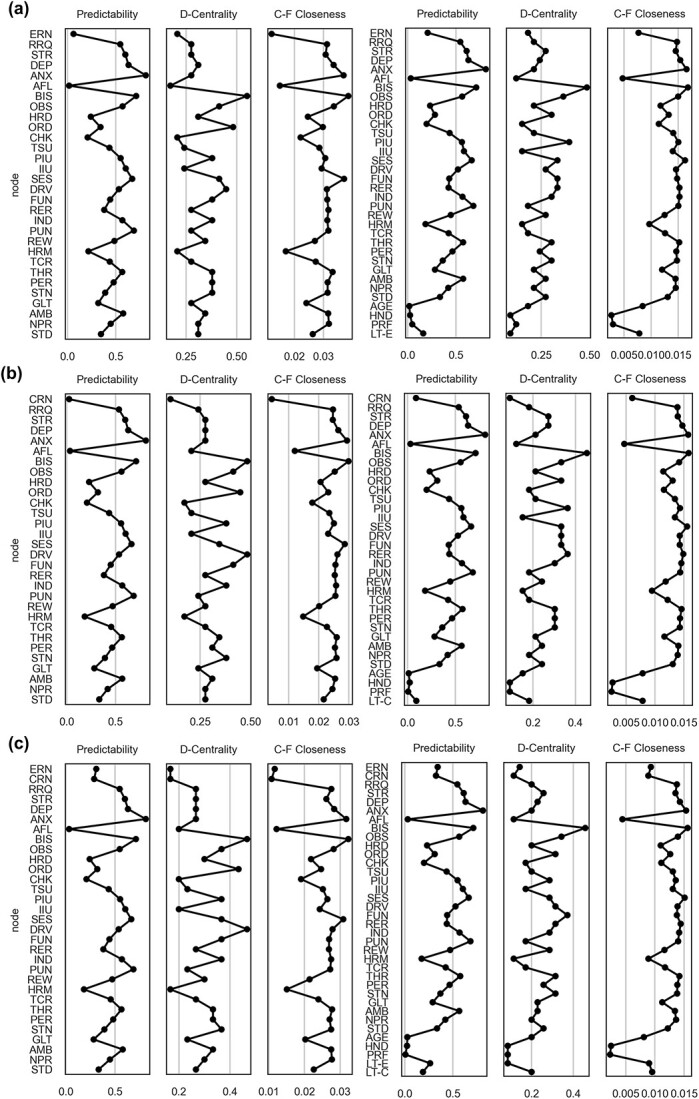
Hold-out predictability, degree centrality (D-Centrality) and current flow closeness centrality (information centrality; C-F Closeness) network measures estimated for the ERN networks (a), CRN networks (b), and networks with both ERN and CRN (c). The left column shows network measures estimated for models without covariates; right panel shows network measures estimated for models with covariates. All node codes are the same as provided in Fig. 3. **Figure source data 1**. Network analysis results: *network_analysis* at https://osf.io/cxkqj/ **Figure source code 2**. https://bit.ly/3TJjISV

From the network measures whose stability was tested, we selected predictability, degree centrality, and current flow closeness centrality (also known as information centrality). All three of these measures offer insights into different aspects of the networks: predictability indicates the best-explained node within the network; degree centrality identifies highly connected nodes that potentially serve as network hubs; information centrality reveals nodes that play crucial roles in mediating information flow within the graph, thus their significance extends beyond a simple count of their connections. All networks had a similar pattern of values for network measures: the trait anxiety yielded the highest hold-out predictability scores (81.20%); the behavioral inhibition node had the highest degree centrality (.45–.55). The information centrality metric incorporated information from both the predictability and degree centrality metrics; the trait anxiety and behavioral inhibition nodes yielded the highest values (.015–.036 and .015–.037, respectively). Fig. 4 shows a summary of the network measures in all six networks.

**Fig. 5:**
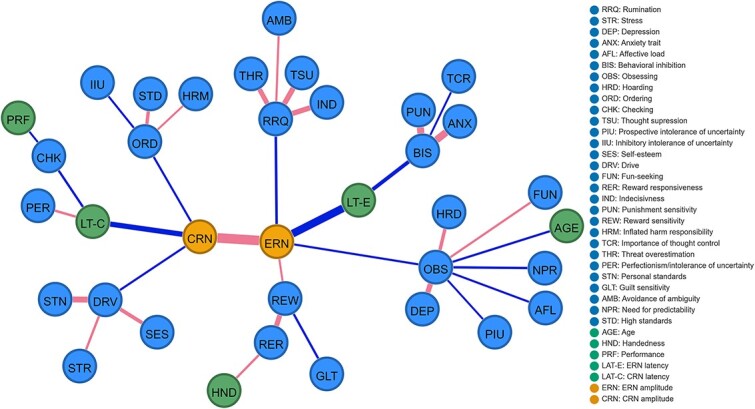
Maximal spanning tree of the ERN network with covariates and CRN control (Fig. 3f).

*Maximal spanning tree*. We estimated a maximal spanning tree of the ERN network with covariates and CRN control (Fig. 3f) to analyze the structure of connections between individual differences included in our analysis from a high-level perspective. The maximal spanning tree reduces the dimensionality of the network and provides a good estimation of its most important paths, thus facilitating high-level analysis of the structure of dense networks. Fig. 5 shows the maximal spanning tree of the ERN network with covariates and with CRN amplitude control.

In the estimated maximal spanning tree, seven distinct clusters of individual traits are visible. The obsessing cluster comprises obsessing, hoarding, depression, affective load, prospective intolerance of uncertainty, need for predictability, the fun-seeking dimension of behavioral activation, and age. The reward cluster encompasses reward sensitivity, the reward responsiveness facet of behavioral activation, guilt sensitivity, and handedness. The anxiety cluster includes behavioral inhibition, trait anxiety, punishment sensitivity, and the importance of thought control. This cluster is connected to the ERN amplitude node through the ERN latency node. The rumination cluster comprises rumination, thought suppression, threat overestimation, avoidance of ambiguity, and indecisiveness. Ordering is grouped with inhibitory intolerance of ambiguity, high standards, and inflated harm responsibility. The drive dimension of behavioral activation forms a cluster with self-esteem, stress, and personal standards. Finally, the last cluster includes checking, performance, and perfectionism/intolerance of uncertainty, and is connected to the CRN amplitude node through the CRN latency node. Among the clusters directly connected to the ERN amplitude node, the strongest connection is with the rumination cluster; the reward and obsessing clusters are connected to the ERN amplitude node with similar strength.

### Stability analyses

#### Network structure stability

**Fig. 6:**
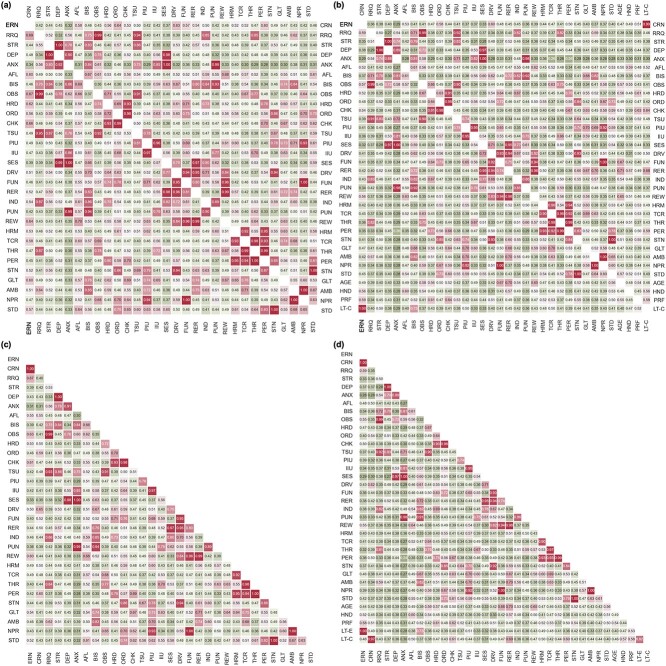
Proportion matrices from the stability selection procedure. The upper triangles show the proportion matrices for CRN; the lower triangles show the proportion matrices for ERN. Panel a represents models without covariates, whereas panel b represents models with covariates. All node codes are the same as provided in Fig. 3. **Figure source data 1**. Support and proportion matrices from the stability selection procedure: *support_proportion* at https://osf.io/cxkqj/

To improve the stability of the networks’ structure, we employed a stability selection procedure, as described in the *Network estimation* section. Fig. 6 displays the proportion matrices derived from the stability selection procedure, illustrating the probabilities of each edge being non-zero across the four estimated network models. Edges that did not exceed the 65% threshold were considered unstable and were thus zeroed in initial support estimates (see Material and Methods).

*Comparison with hold-out dataset*. We tested whether the estimated networks’ structures were stable across different populations. We compared the adjacency matrices of networks that were estimated on the training and testing sets using Pearson’s *r* correlation coefficient and Hamming distance. All networks estimated on the training set displayed significant similarity to those estimated on the testing dataset. The correlations between models were .22 for ERN models without covariates, .22 for ERN models with covariates, .25 for CRN models without covariates, .24 for CRN models with covariates, .23 for ERN model with CRN control, and .25 for ERN models with covariates and CRN control (all $P$ <.001). The Hamming distance-based similarities were as follows: .65 for ERN models without covariates, .69 for ERN models with covariates, .67 for CRN models without covariates, .70 for CRN models with covariates, .67 for ERN model with CRN control, and .71 for ERN models with covariates and CRN control.

As the relationships between ERP amplitudes and individual differences were our main focus, we further estimated the stability of these associations across different populations. We compared one-dimensional adjacency matrices representing only ERN/CRN association structures. However, it is worth noting that the comparison of small *binary* vectors ($\sim $ 30 elements) using Pearson’s *r* should be interpreted with caution. The Hamming distance metric is a more reliable measure of the similarity between two models. The correlations between ERN associations in models were .39 (*P* = .034) for ERN models without covariates, .28 (*P* = .108) for ERN models with covariates, .45 (*P* = .009) for ERN models with CRN control, and .34 (*P* = .039) for ERN models with covariates and CRN control. In turn, the Hamming distance-based similarities were .80 for ERN models without covariates, .76 for ERN models with covariates, .81 for ERN models with CRN control, and .81 for ERN models with covariates and CRN control. The correlations between CRN associations in models were .05 (*P* = .796) for CRN models without covariates, .27 (*P* = .129) for CRN models with covariates, .27 (*P* = .129) for models with ERN control, and .54 ($P$ < .001) for models with covariates and ERN control. The Hamming distance-based similarities were .73 for CRN models without covariates, .80 for CRN models with covariates, .79 for models with ERN control, and .89 for models with covariates and ERN control.

These results suggest that the ERN model with covariates and CRN control was the most stable and produced the most consistent ERN/CRN–individual traits associations between training and testing sets.

*Sensitivity and specificity*. To further assess the stability of the created networks, we calculated the sensitivity (true positive rate) and specificity (true negative rate) of the edges using 1,000 bootstrap samples.

In the ERN network without covariates, all estimated associations with ERN exhibited reasonably high sensitivity (.69–.81). The lowest sensitivity had associations between ERN amplitude and ordering (.68) and checking (.69). The ERN associations with the lowest specificity (thus most likely to be false negatives) included affective load (.25), thought control (.27), and guilt sensitivity (.32). After controlling for covariates, the sensitivity of estimated ERN associations slightly dropped (.73, .70, .74 for rumination, obsessing, and reward sensitivity, respectively). The sensitivity of the new association between ERN and avoidance of ambiguity was .34, indicating that this association lacks stability and reliability. This may explain the absence of a significant difference in the bootstrapped edge-weights test between the ERN network and the ERN network with covariates. The specificity of the associations that disappeared after controlling for covariates was .35, .43, and .37, further suggesting that these associations have low reliability in being true negatives. Furthermore, when additionally controlling for the shared variance with CRN, the sensitivity for rumination, obsessing, and reward sensitivity was reasonably high (.75, .72, and .73 for rumination, obsessing, and reward sensitivity, respectively). The lowest specificity was observed in the links between ERN amplitude and performance (.10), handedness (.30), drive (.38), hoarding (.46), and affective load (.32). The specificity of the links between ERN and ordering and checking increased to .72 and .53, respectively; this suggests that controlling for the variance shared with CRN improved the specificity of the results. Notably, the specificity of the link between ERN amplitude and trait anxiety, as well as behavioral inhibition, was consistently high at .86 and .61, respectively, indicating a true lack of association between ERN amplitude and both trait anxiety and behavioral inhibition in our sample.

In the CRN network without covariates, associations with CRN exhibited high sensitivity (.68, .75, .76 for perfectionism/intolerance of uncertainty, the drive dimension of behavioral activation, and ordering, respectively). Of the CRN associations, the lowest specificities were observed for affective load (.23), hoarding (.30), and fun-seeking (.31). When controlling for covariates, the sensitivities of CRN links remained similar (.79 for the drive dimension of behavioral activation and .69 for ordering). The specificity of the link between CRN amplitude and perfectionism/intolerance of uncertainty, which disappeared after covariates adjustment, was .58; this suggests a high probability that this association is a true negative. When controlling for the variance shared with ERN, the sensitivity of the link between CRN amplitude and ordering dropped to .62, while the sensitivity of the link with the drive dimension of behavioral activation increased to .88, indicating that the association between the drive and CRN amplitude is particularly robust. The lowest specificity was observed for affective load (.44), fun-seeking (.44), and performance (.23).

Overall, the results of the sensitivity and specificity tests suggest that the estimated associations between ERN/CRN amplitudes and individual traits are stable and robust, whereas the estimated lack of associations is less reliable, particularly for affective load and performance. The results of the sensitivity and specificity analyses can be found in supplementary materials.

*Edge-weight stability*. All estimated networks had high edge-weight stability; in most cases, the width of 95% confidence intervals for the partial correlation coefficients was around .02. The edge-weight stability was calculated using 1000 bootstrapped samples. The average values of edges’ weights along with bootstrapped 95% confidence intervals can be found in Supplementary Materials.

#### Network measures stability

We tested selected network measures for their stability to ensure that the measures we are reporting are meaningful. We decided to test the predictability, degree centrality, closeness, betweenness, current flow closeness centrality, and current flow betweenness centrality measures for their stability (see *Network estimation* section for more details). Predictability yielded excellent stability, with a mean correlation of .96 between predictability estimated on the original sample and predictability estimated on the bootstrap samples that consisted of 30% of the original dataset. The measures of closeness and betweenness based on current flow exhibited significantly higher stability than their classical implementations. Degree centrality and closeness both had low stability, with the mean correlation between the measures estimated on the original sample and those estimated on the bootstrap subsamples reaching .40 when the subsamples consisted of 50% of the original dataset. Betweenness had the lowest stability; the mean correlations between betweenness estimated on the original data and betweenness estimated on the bootstrap subsamples were lower than .10 for most sizes of subsamples. The results of the network measures stability analyses can be found in supplementary materials.

## Discussion

Prior research on individual differences and error monitoring have yielded inconsistent results regarding the relationship between ERN amplitude and affect- and motivation-related individual differences. Our study is the first attempt to capture the relationships among ERN amplitude, multiple individual differences, and demographic covariates simultaneously; it is also the first to explore which relationships are direct and which are more likely to be mediated, and to explore the interplay between affective and motivational individual differences and their potentially joint impact on error monitoring.

The mean ERN amplitude node was negatively associated with rumination, ordering, and obsessing; it was also positively associated with OCD hoarding and checking symptoms, and reward sensitivity. When corrected for age, handedness, ERN latency and performance, the new relationship between ERN and avoidance of ambiguity emerged, and the relationships between ERN and OCD hoarding, checking, and ordering symptoms disappeared. Instead, ERN amplitude shared a strong edge with ERN latency; ERN latency also shared a negative edge with behavioral inhibition and a positive edge with thought control. After adjusting for mean CRN amplitude, more pronounced ERN amplitudes were associated with higher levels of rumination and obsessing, and lower level of reward sensitivity. Thus, controlling for both CRN amplitude as well as age, handedness, ERN latency and performance changed associations between ERN amplitude as individual traits.

### Covariates

*ERN Latency*. Multiple studies suggest that ERN latency reflects the effectiveness of response control, with earlier ERN peaks indicating a higher likelihood of error correction. Research has found that ERN latency is shorter for partial errors than for full errors (Masaki et al. 2004; Carbonnell and Falkenstein 2006; Roger et al. 2014; Ficarella et al. 2019), and for errors that are corrected compared to those that remain uncorrected (Fiehler et al. 2005; Hoffmann and Falkenstein 2010), even though the amplitude does not differ in these cases. Additionally, more difficult tasks tend to result in longer ERN latencies (Falkenstein 2004). In sequential tasks, in which participants begin to learn over the course of the task, ERN latency is usually shorter (Paas et al. 2021) and often decreases over time (Beaulieu et al. 2014). Conversely, there has been limited research on the associations between ERN latency and individual differences. Olvet and Hajcak (2009b) reported more pronounced ERN latencies in women than in men. Meyer and Gawlowska (2017) observed that individuals with high trait anxiety show an earlier ERN when errors are punished. Further, prolonged ERN latencies were reported in participants with OCD diagnoses compared to healthy controls (Johannes et al. 2001).

In our study, longer ERN latencies were initially associated with higher levels of prospective intolerance of uncertainty and lower levels of behavioral inhibition. However, after adjusting for CRN amplitude and latency variance, longer ERN latencies were linked only to lower levels of behavioral inhibition. Since prospective intolerance of uncertainty was found to be closely related to obsessing symptoms in our analysis, our results appear to support the findings of Johannes et al. 2001.

As for behavioral inhibition, according to Gray’s model (Gray and McNaughton 2000) it is conceptualized as an attentional system for evaluating a potential response conflict or threat. It aims to interrupt ongoing behavior in order to facilitate the processing of punishment, nonreward, or novelty in preparation of subsequent actions. The role of the behavioral inhibition system is conceptually similar to the conflict-monitoring function of the ACC, which is sensitive to unexpected events and conflicts between competing responses. Thus, considering response conflict monitoring theory (Carter et al. 1998), the negative relationship between behavioral inhibition and ERN latency indicates that the conflict between competing response tendencies culminates more quickly in individuals with a higher level of behavioral inhibition. Alternatively, in line with reinforcement learning theory (Holroyd and Coles 2002) or mismatch theory (Gehring et al. 1993; Falkenstein et al. 2000), our results point to faster detection of expectancy violation or a mismatch between the actual response and the desired state. On the neural level, the faster error detection in individuals higher in behavioral inhibition is probably caused by noradrenergic effects. Noradrenergic fibers stemming from the locus coeruleus project to the ACC, providing complementary modulation to this region alongside dopaminergic input (Joshi and Gold 2022). The release of noradrenaline by the locus coeruleus enhances the processing of threatening stimuli or events (such as errors) by increasing the signal-to-noise ratio in cortical areas involved in vigilance, attention, and decision-making (Nieuwenhuis et al. 2005). This mechanism may facilitate faster error detection through a quicker onset of error-related neural activity. It is worth noting that a similar association between attention, arousal, and ERP latencies was also observed for inhibition-related ERPs (Reza et al. 2007; Howe 2014; Marin et al. 2021). Intriguingly, faster error detection aligns with predictive processing frameworks, which suggest that a “better-safe-than-sorry” processing strategy could be a general vulnerability factor for negative affect, including enhanced behavioral inhibition (Van den Bergh et al. 2021). Specifically, this strategy involves a speeded information processing heuristic characterized by reduced sensory detail, allowing threat-related categorical priors to dominate experience. This hypothesis is further reinforced by the findings of Meyer and Gawlowska (2017), who reported that individuals with high trait anxiety exhibit shorter ERN latencies when errors are punished.

The lack of an association between ERP latency and behavioral inhibition in the CRN network suggests that this change in the length of the response monitoring process is specifically related to response processing that ends with an incorrect response.

We did not find the relationship between performance and the ERN latency, as would be suggested by studies on error corrections. However, it is possible that the ERN’s effect on performance would be more immediate, and that it would affect performance on the next trial (i.e. post-error adjustments in behavior), rather than boost overall task accuracy.

*Performance*. In both the ERN and CRN network (Fig. 3b, d), better performance was associated with higher levels of OCD hoarding and personal standards and lower levels of OCD checking. In the network that includes both ERN and CRN amplitude nodes, this pattern of associations remained unchanged. The negative association between checking and performance suggests that checking is, in fact, a non-adaptive strategy that does not lead to better performance, at least not in the experimental environment. Contrary to our expectations, performance was not associated with the ERN amplitude node nor the CRN amplitude node. Previous studies present conflicting evidence on the relationship between ERN amplitudes and overall performance in the task. Some studies have found that larger ERN amplitudes predict better accuracy (e.g. Gehring et al. 1993), while others have not observed this relationship (Fiehler et al. 2005). As noted earlier, the association between ERN and performance may be limited to intra-individual effects rather than inter-individual differences. Therefore, single-trial analysis, similar to that used by Mattes et al. (2022), would be better suited to measure the effect of ERN latency on subsequent performance. Additionally, it has been shown that ERN amplitude decreases with an increasing number of committed errors (Suchan et al. 2018). Given that performance measures include information about the number of errors made, we anticipated a correlation between performance and the mean amplitude of ERP components; however, this association was not present.

*Age*. In the ERN network (Fig. 3b), age shared a moderate negative link with the reward responsiveness and fun-seeking dimensions of behavioral activation, reward sensitivity, indecisiveness and the OCD obsessing and hoarding symptoms. Exactly the same pattern of relationships was present in the CRN network (Fig. 3d) and ERN network with control for CRN (Fig. 3f). Age did not share a link with ERN or CRN amplitude. These results only partially align with the existing findings. ERN is known to be increased with age, especially in children and adolescents (see Boen et al. 2022, for a review). The relationship between ERN and age in adults is less studies. For example, Riesel et al. (2023) reported a significant relationship between ERN amplitude and age, but no association between CRN amplitude and age. The discrepancy between results of Riesel et al. (2023) and ours may be due to the greater age variability in Riesel et al.’s sample compared to ours. In another line of research, Eppinger et al. (2008) reported that the association between ERN and age disappeared after controlling for performance. However, our correlation analysis (see online supplementary materials) also showed no relationship between ERN and age. Therefore, we attribute the lack of a link between ERN and age in our study to the limited age variability in our sample. Our results do not preclude a relationship between ERN amplitude and age, especially one related to developmental changes in ERN amplitude from childhood to adulthood (e.g. Chong and Meyer 2019; Chong et al. 2020; Mehra et al. 2022).

*Handedness*. Handedness shared a link with affective load, the reward responsiveness facet of behavioral activation, and threat overestimation, in all three the ERN (Fig. 3b), the CRN (Fig. 3d), and the ERN with CRN control (Fig. 3f) networks. The handedness node did not share a link with ERN or CRN amplitude, thus suggesting that differences in brain structural anatomy resulting from different handedness (Sha et al. 2021; see also Polich and Hoffman 1998) do not necessarily result in different response-related ERPs amplitudes. Wright and Hardie (2015) reported associations between reward processing, indecisiveness, and handedness, but these associations were limited to interaction effects: handedness moderated the relationship between reward responsiveness and indecisiveness. Our study seems to partially confirm the findings of Wright and Hardie, demonstrating the link between handedness and reward responsiveness.

### ERN and reward, punishment, threat, and anxiety

*Reward sensitivity*. Drawing on reinforcement learning theory, the Research Domain Criteria framework (RDoC; Cuthbert 2014), which aims to define and link fundamental biological and behavioral components of normal and abnormal functioning, suggests that ERN amplitude may serve as a biomarker of reward learning. Although the conceptual link between ERN and the reinforcement learning construct seems to be clear as ERN reflects the error monitoring process and the ability to monitor and evaluate mistakes should therefore enable us to learn from them (Sutton and Barto 1998), it is not yet clear how exactly ERN amplitude, rewards, and behavioral adaptation are interrelated. In our study, more pronounced ERN amplitude was linked to lower levels of reward sensitivity; this was also present after adjustment of covariates and CRN. This result extend previous research, and reconfirms that ERN amplitude is sensitive to reward-motivated behavior (see Boksem et al. 2008; Santesso and Segalowitz 2009). These results are usually explained by reduced ACC activity associated with increased sensitivity to reward. However, such results do not necessarily imply the utility of ERN amplitude as a biomarker of reward learning. According to Weinberg et al. (2016), ERN amplitude might reflect the magnitude of the early response evaluative stage, which only triggers downstream processes that may or may not lead to adaptive behavioral adjustments. Further research using, e.g. source modeling is needed to clarify whether reward sensitivity affects only early error evaluation or also behavioral adaptation, and whether ERN amplitude reflects only the evaluative stage or also compensatory behaviors. Until then, the validity of ERN as a marker of reward learning cannot be definitively determined.

Although ERN amplitude shared a unique link with reward sensitivity, neither ERN nor CRN shared a link with the reward responsiveness facet of behavioral activation; this suggests that reward sensitivity and the reward responsiveness facets of behavioral activation, although closely related, are separate constructs. Our methodology allowed us to identify both unique and shared neighbors of reward sensitivity and reward responsiveness, facilitating a deeper exploration of their characteristics. Age, along with the drive and fun-seeking facets of behavioral activation, emerged as common factors influencing both reward sensitivity and reward responsiveness; specifically, higher levels of reward sensitivity/responsiveness were associated with increased drive and fun-seeking, and decreased age. Reward sensitivity showed unique positive connections with affective load, threat overestimation, and prospective intolerance of uncertainty, and negative connections with behavioral inhibition and guilt sensitivity, whereas reward responsiveness was positively associated with avoidance of ambiguity, handedness, stress, behavioral inhibition, guilt sensitivity, and self-esteem. Further, reward sensitivity shared the strongest link with fun-seeking, while the reward responsiveness facet of behavioral inhibition shared the strongest link with self-esteem. These distinct associations support the notion that reward sensitivity and reward responsiveness are separate constructs: reward sensitivity describes the extent to which an individual’s behavior is directed toward competition and is motivated by risk-taking and sensation-seeking (Torrubia et al. 2001; Santesso and Segalowitz 2009); conversely, reward responsiveness refers to the degree of *positive* reactions to rewards, regardless of competitive circumstances (Carver and White 1994). The distinct association between ERN amplitude and reward sensitivity, alongside the absence of any association with reward responsiveness, suggest that less pronounced ERN amplitudes are specifically associated with reduced sensation-seeking and risk-taking behaviors.

*Sustained Threat*. ERN has been proposed to be a biomarker of Sustained Threat in RDoC’s Negative Valence System domain. Indeed, an important role of the ACC, which is often identified as a neural generator of ERN, is to integrate information about punishment to guide behavior (Cavanagh and Shackman 2015). Thus, we expected ERN amplitude to be associated with punishment sensitivity. However, we did not find a direct association between punishment sensitivity and ERN amplitude; also, we did not find that ERN amplitude was related to depression, stress, overestimation of threat, or OCD symptoms other than obsessing, which are usually linked to punishment-guided behaviors. Instead, punishment sensitivity, depression, and stress were associated with ERN amplitude through behavioral inhibition and ERN latency. Boksem et al. (2008) reported an association between punishment sensitivity, measured with behavioral inhibition, and ERN amplitude, but only in the punishment condition. It is possible that the relationship between punishment sensitivity and ERN is more complex than previously assumed and does not reveal itself under all experimental conditions. In addition, we quantified ERN as a mean time-window amplitude, while Boksem et al. quantified it as a mean amplitude around the ERN peak. On the other hand, Santesso and Segalowitz (2009) did not observed any associations between ERN amplitude and punishment sensitivity, as measured by the Sensitivity to Punishment and Sensitivity to Reward Questionnaire (SPSRQ; Torrubia et al. 2001). Further studies should investigate the relationship between various measures of punishment sensitivity, ERN latency, and ERN amplitude quantified in different ways.

In addition to punishment sensitivity, the constructs listed in the RDoC framework as behavioral measures of sustained threat include anhedonia/decreased appetitive behavior, anxious arousal, attentional bias to threat, avoidance, helplessness behavior, increased conflict detection, and increased perseverative behavior. Based on a large-scale study in health adolescence, Weinberg et al. (2016) proposed a more specific relation between sustained threat and ERN, suggesting that ERN might reflect abnormal error control aimed at avoiding negative situations that could lead to threatening errors. Weinberg et al. supported their claims by showing the association between ERN amplitude and the OCD checking symptom, which is clearly abnormal error control behavior. However, in our study, we found a direct link between ERN amplitude and checking only in the ERN model without covariates control (Fig. 3a); this association disappeared after adjusting for covariates. Instead, we observed that checking was associated with performance, and had an indirect link to ERN amplitude through hoarding and obsessing, which, in turn, showed a strong association with ERN amplitude. Although we did not confirm the association between checking and ERN amplitude, our results support the hypothesis that ERN amplitude is linked to sustained threat, as defined by the RDoC framework. In contrast, Riesel et al. (2023) found no association between the compulsivity latent factor, loaded by OCD symptoms, and ERN amplitude. However, in their study, OCD obsessing loaded more strongly onto the anxious misery factor than the compulsivity factor, which was, in turn, associated with ERN amplitude. Given that in our study, obsessing was strongly connected with depression, threat overestimation, thought suppression, and the need for predictability, we attribute the association between obsessing and ERN amplitude to threat sensitivity or anxiety rather than to OCD compulsivity measures.

In our models, ERN amplitude was strongly associated with rumination. While rumination is not typically discussed as a symptom of sustained threat, it is undeniably linked to punishment sensitivity (Aldao and Nolen-Hoeksema 2010; Izadpanah et al. 2017; Khoshfetrat et al. 2022) as well as avoidance behaviors aimed at abnormal error control (Smith and Alloy 2009), which has been shown to co-occur with increased ERN amplitude multiple times (Hajcak and Foti 2008; Olvet and Hajcak 2008; Zambrano-Vazquez et al. 2019). The direct and robust association between ERN amplitude and rumination in our study appears to confirm the connection between avoidance of negative situations, abnormal error control, and ERN amplitude.

Taken together, our findings suggest that a key factor in sustained threat and punishment sensitivity, as well as in their association with ERN amplitude, may be an (abnormal) need for cognitive and/or behavioral control to avoid negative situations that could result in threatening errors.

*Anxiety*. A substantial body of evidence indicates that anxiety symptoms are associated with increased ERN amplitude. Among the various facets of anxiety, anxious apprehension/worry is the most frequently mentioned aspect that is linked to ERN amplitude. Anxious apprehension, characterized by persistent worrying and avoidance behaviors, is typically evaluated using measures of OCD or behavioral inhibition (see Moser et al. 2013). Additionally, a number of studies have also reported an association between trait anxiety and ERN amplitude. Recently, Riesel et al. (2023) showed the association between ERN amplitude and the anxious misery latent factor, loaded by the OCD symptoms, trait anxiety, worry, and neuroticism. However, in our study we did not find any association between ERN amplitude and behavioral inhibition, trait anxiety, or other closely related measures, such as inhibitory intolerance of uncertainty, stress, or affective load. Similar results in a non-clinical sample were reported by Härpfer et al. (2020), Muir et al. (2020), and Seow et al. (2020), suggesting, that the association between ERN amplitude and anxiety-related symptoms in a non-clinical population might be much smaller than previously assumed. These results are further supported by the mass-univariate statistics conducted by Chen and Itier (2024), which revealed lack of association between ERN amplitude and anxious traits in non-clinical individuals with subclinical anxiety, and the multiverse analysis of the ERN–anxiety relationship by Clayson (2024), which reported that the association between ERN and anxiety in a general population is weak. Thus, the absence of a significant association between ERN amplitude and trait anxiety in our study may be attributed to the non-clinical status of our population.

Simultaneously, all anxiety measures, i.e. such as behavioral inhibition, trait anxiety, punishment sensitivity, and inhibitory intolerance of uncertainty, were strongly interconnected. These measures were linked to the ERN amplitude node through rumination and behavioral inhibition, and through ERN latency and behavioral inhibition when controlling for covariates. These results offer new insight into the relationship between anxiety and ERN amplitude in non-clinical samples, suggesting that rumination is more specifically associated with ERN than trait anxiety or behavioral inhibition in these groups. Future studies should investigate the association between rumination, anxiety, and ERN in both clinical and non-clinical samples.

As previously mentioned, there is robust evidence that anxious apprehension comprises a range of narrower symptoms, such as worry, negative affect, or sensitivity to threat (Olatunji et al. 2010; Sharp et al. 2015). The centrality measures within the constructed networks further validate this notion: behavioral inhibition and trait anxiety were the most central nodes in all ERN and CRN networks, thus indicating that they are multidimensional constructs that include many narrower symptoms. Given that ERN is likely uniquely associated with only certain components of anxiety, it becomes clear that in some situations a direct relationship between anxious apprehension and ERN, and between trait anxiety and ERN, cannot be observed when controlling for all other constructs included in the networks. Our findings imply that in many cases the apparent association between ERN amplitude and trait anxiety is likely spurious and should be attributed to other symptoms that are also captured by trait anxiety and behavioral inhibition. Furthermore, fluctuations in anxious apprehension/trait anxiety levels may stem from various symptoms in such cases, ranging from self-esteem issues and depression to thought suppression. Our findings raise doubts about the utility of trait anxiety and behavioral inhibition measures in studying the relationship between anxiety, threat, and ERN, especially in non-clinical samples with low severity of anxiety symptoms, suggesting more dimension-oriented approach, as employed by Riesel et al. (2023) or Seow et al. (2020).

### OCD symptoms

The relationship between ERN and CRN amplitudes and OCD symptoms has been well-documented over time. A recent meta-analysis by Riesel (2019) confirmed a robust association between more pronounced ERN amplitudes and OCD diagnosis. In our study, we found that more pronounced ERN amplitudes were linked to lower levels of checking and hoarding and higher levels of obsessing and ordering. However, when controlling for ERN latency, age, handedness, and performance, only the association with obsessing remained significant. These findings partially align with those of Riesel et al. (2023), who reported an association between increased ERN amplitudes and higher OCD severity when controlling only for age and gender. In a dimensional analysis, Riesel et al. observed that the relationship between compulsivity and ERN amplitudes disappeared, when controlling for anxiety symptoms and CRN amplitudes. Similarly, Seow et al. (2020) found no association between the compulsive behavior and intrusive thought factor and ERN amplitude. Our findings suggest that the relationship between ERN amplitude and OCD symptoms might be highly specific and limited to only certain OCD symptoms, particularly obsessing (see Riesel et al. 2019, Supplementary Table 5), as well as sensitive to covariates. This might explain the contradictory results found in factor analyses. Further, performance proved to be a particularly influential covariate in the relationship between ERN and OCD symptoms. After adjusting for covariates, higher levels of checking were associated with lower performance, while higher levels of hoarding were linked to better performance. These findings suggest that the association between ERN amplitude and OCD checking symptoms may be especially sensitive to performance factors, possibly reflecting the reduced ERN amplitude observed with an increased number of errors (Suchan et al. 2018).

Contrary to our expectations, the CRN amplitude was consistently associated only with OCD ordering. As CRN amplitude has been suggested to reflect the elevated tendency to check response correctness (Falkenstein et al. 2000; Vidal et al. 2000), we expected association between ERN and checking/obsessing OCD symptoms. To date, there is limited research on the relationship between CRN amplitude and OCD symptoms, and the existing findings are mixed. For instance, Riesel et al. (2023) found that more pronounced CRN amplitudes were related to higher levels of compulsivity and personal standards factors. In contrast, Riesel et al. (2015) and Riesel et al. (2019) reported no significant association between CRN amplitudes and OCD symptoms severity. Our results suggest that the relationship between OCD symptom severity and CRN amplitude, if it exists, may be weak and challenging to detect in a non-clinical population.

### Correct-related negativity

In all networks more pronounced CRN amplitudes were robustly associated with higher levels of ordering and the drive dimension of behavioral activation. Initially, more pronounced CRN amplitudes were also related to higher levels of perfectionism/intolerance of uncertainty; however, after adjusting for covariates, perfectionism/intolerance of uncertainty shared an unique link with the CRN latency node. These results contradict those of Riesel et al. (2023), who found no association between CRN and conscientiousness or positive affect factors, which was loaded by the drive dimension of behavioral activation. Given that the drive facet of behavioral activation reflects the motivation to pursue one’s goals (Carver and White 1994), our findings suggest that increased CRN amplitude might be associated with heightened attention and vigilance, as well as increased motivation to respond correctly (see Noordt et al. 2015; Imhof and Rüsseler 2019; Files et al. 2021).

CRN amplitudes were not associated with either age or performance. This finding aligns with Riesel et al. (2023), who also reported no significant link between CRN amplitudes and age. However, Riesel et al. (2019) found that older participants exhibited more pronounced CRN amplitudes. Given that only a limited number of studies have explored the associations between CRN amplitudes and covariates like age, there is still far from a consensus on the developmental trajectory of CRN and its connections to performance.

### Limitations

This work has certain limitations, the most important of which is the limited variability of the analyzed symptoms, especially highly clinical ones such as OCD symptoms, as participants with any mental disorder were excluded. Although ERN is generally expected to be associated with individual differences, especially anxiety- and reward-related symptoms, regardless of their clinical severity, our dataset did not cover the entire spectrum of severity of some symptoms, thus it inherently limits the clinical conclusions in two ways: (1) it is possible that the patterns of associations between ERN and some symptoms are different in the clinical population; (2) our analysis may not have captured some of the important clinical relationships due to the overall low levels of symptoms’ severity in the healthy volunteer population. As shown by recent meta-analysis by Saunders and Inzlicht (2020), association between anxiety and ERN amplitude in non-clinical sample is weak. To be clinically relevant, the study needs to be replicated in a clinical population. Moreover, we did not control for gender, even though ERN is known to be gender-sensitive. Our models required a Gaussian distribution of the variables to meet the assumptions of Gaussian Graphical Models (GGMs). GGMs assume that data follow a multivariate normal distribution because they rely on the covariance/precision matrix, which is applicable to continuous variables that are normally distributed. Binary variables, by contrast, have limited covariance interpretation because they take on only two values. This complicates the direct use of covariance to describe their relationships with continuous variables and makes it difficult to model reliable conditional dependencies. To explore the role of gender in associations between ERN and individual differences, future studies could use Mixed Gaussian Graphical Models, which facilitate modeling the interaction effects of categorical variables (Haslbeck and Waldorp 2020). Furthermore, women tend to have higher levels of anxiety-related symptoms than men, as was also reflected in our dataset. When the variance of a variable differs between genders, adjusting for gender might falsely suggest that gender itself is associated with ERPs. In reality, these associations may be driven by differences in variability rather than genuine gender effects. Future studies should try to replicate the presented results on gender groups that do not differ significantly in symptom intensity. Finally, the estimated networks had many nodes, thus their accuracy even on a sample that consists of 236 individuals is limited. Despite the networks’ high accuracy and stability estimates, it is possible that some weak associations were not detected. The presented study will benefit from replication on a larger sample in order to test the plausibility of the conclusions.

## Conclusions

Despite substantial efforts to explain the association between error-related brain activity and factors related to reward, punishment, threat, motivation, and anxiety, our understanding of changes in ERN amplitude remains limited. Research investigating associations between ERN and individual differences has yielded inconsistent results. In our study, we sought to contribute to this ongoing debate by adopting a network perspective which highlights the potential complexity of the links between ERN and symptoms, thus emphasizing the system-like dynamics of interactions among different variables. We found that neither trait anxiety nor anxious apprehension measured as behavioral inhibition were associated with ERN amplitude in our non-clinical sample. This finding aligns with the recently emerging view of a fragile connection between trait anxiety and ERN in non-clinical populations. Instead, ERN was uniquely associated with rumination and obsessing. It was also associated with reward sensitivity. These results support the thesis that ERN amplitude reflects abnormal cognitive and behavioral control aimed at avoiding negative or uncertain situations that might result in threatening errors. Trait anxiety and behavioral inhibition were the most central nodes in the constructed networks, thus indicating that they are multidimensional constructs that comprise various narrower symptoms.

Our analyses emphasize that changes in ERN amplitude are associated with specific narrow dimensions of anxiety. Moreover, they suggest that some apparent links between ERN amplitude and individual differences may be spurious and potentially result from shared common causes. We hope that our findings will contribute to a better understanding of the role that symptoms related to anxiety, reward, and punishment play in modulating error-related brain activity.

## Supplementary Material

supplementary_materials_bhae397
