# Supplementary material for: A network analysis of affective and motivational individual differences and error monitoring in a non-clinical sample
Source: Cereb Cortex. 2024 Oct 25;34(10):bhae397. doi: 10.1093/cercor/bhae397 (PMC11513196; doi:10.1093/cercor/bhae397)
Supplement: supplementary_materials_bhae397 [file supplementary_materials_bhae397.pdf]

Distributions of questionnaire measures, covariates, and ERP amplitudes in the training and testing dataset

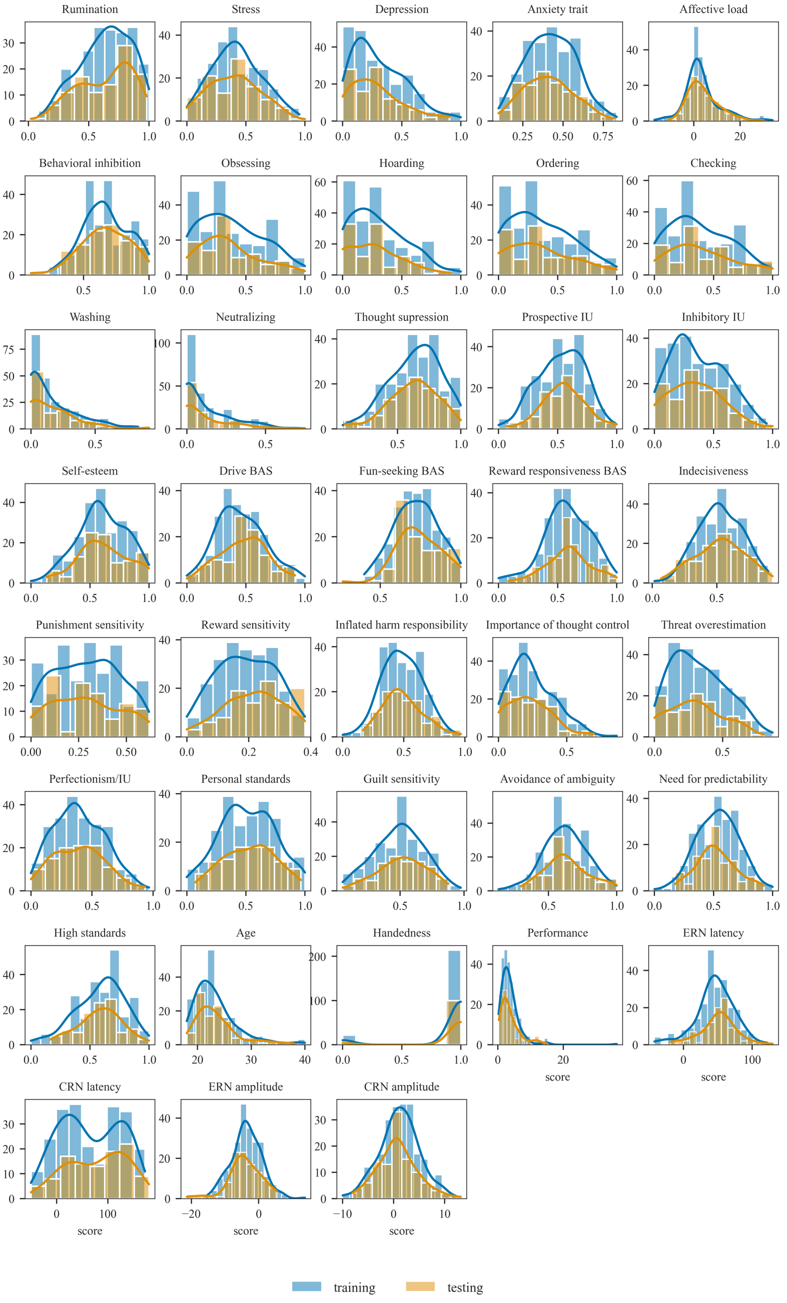

Fig. 1. Distributions of questionnaire scores in the training (blue) and testing (yellow) dataset. The questionnaire scores are normalized such that 0 corresponds to the smallest and 1 to the largest possible value that can be obtained on a given scale. There were no statistical differences between the training and testing datasets in any scores. Questionnaires used to collect self-report measures are described in the Materials and Methods section. IU = intolerance of uncertainty; BAS = behavioral activation; Performance = ratio of inhibited to uninhibited no-go responses; Handedness = self-declared, 1 corresponds to right-handedness, 0 corresponds to left-handedness; ERN = error-related negativity; ERN amplitude = mean ERN amplitude at Fz (0-100 ms); ERN latency = 50% fractional area latency of ERN; CRN = correct-related negativity; CRN amplitude = mean CRN amplitude at Fz (0-100 ms); CRN latency = 50% fractional area latency of CRN.

The sensitivity and specificity estimates of the network models

Sensitivity and specificity estimates help evaluate the effectiveness of the applied network estimation method in capturing the true underlying network structure. An edge's sensitivity measures the proportion of true positives, i.e., the proportion of cases in which an edge was correctly identified as present by the network estimation method. An edge's specificity measures the proportion of true negatives, i.e., the proportion of cases in which an edge was correctly identified as absent by the network estimation method. Higher sensitivity values for edges present in the true network and higher specificity values for edges absent in the true network indicate a more robust network estimation method and a more reliable estimated network structure. We utilized a bootstrapping procedure to assess the sensitivity and specificity of the edges. This involved estimating 1000 networks based on bootstrapped samples; then, we calculated how often the edge present in our original network was present in the bootstrapped networks (sensitivity) and how often the edge absent in our original network was absent in the bootstrapped networks (specificity). The full results on specificity and sensitivity can be found in *structure_stability_estimates/sensitivity_specificity* directory at `https://bit.ly/3Vn8WTk`.

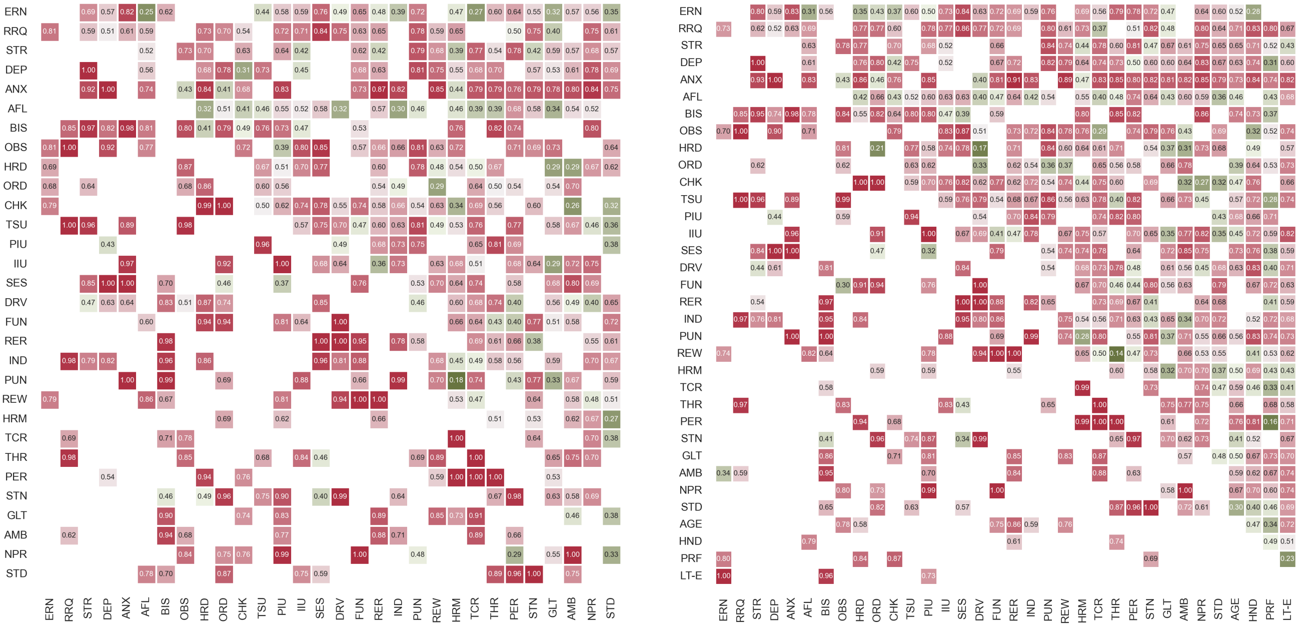

(a) The ERN network

(b) The ERN network with covariates

Fig. 2. Results of sensitivity and specificity tests of the ERN with covariates model. The upper triangles show the specificity results; the lower triangles show the sensitivity results.

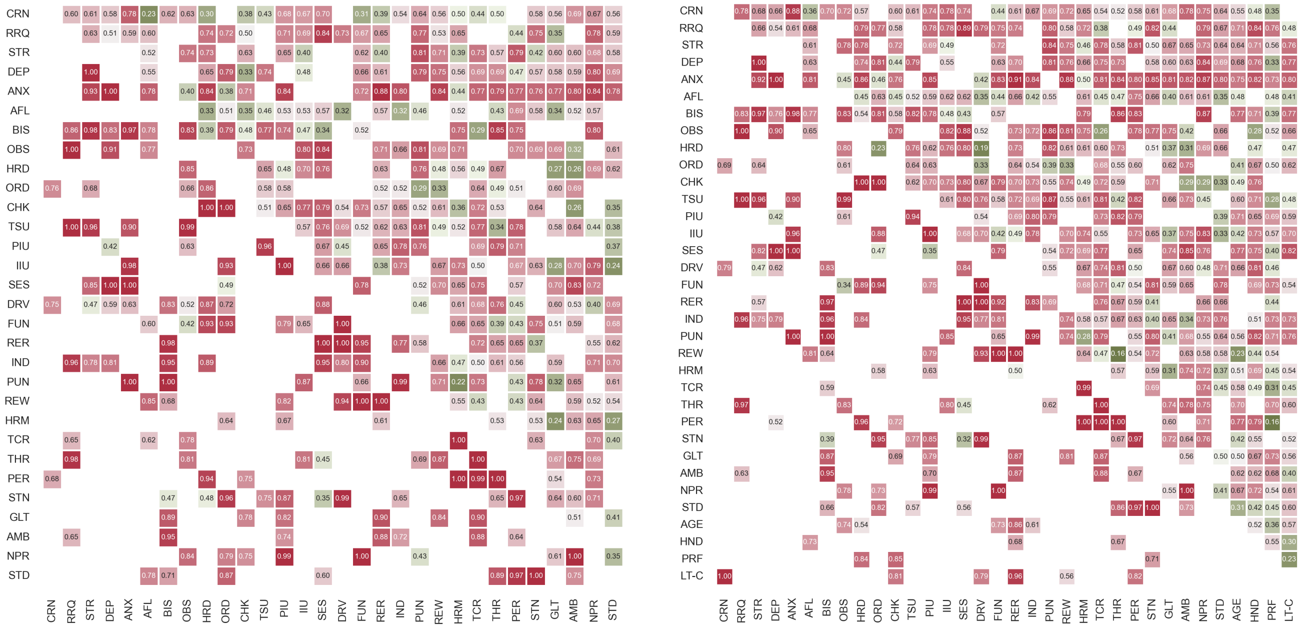

(a) The CRN network (b) The CRN network with covariates

Fig. 3. Results of sensitivity and specificity tests of the CRN with covariates model. The upper triangles show the specificity results; the lower triangles show the sensitivity results.

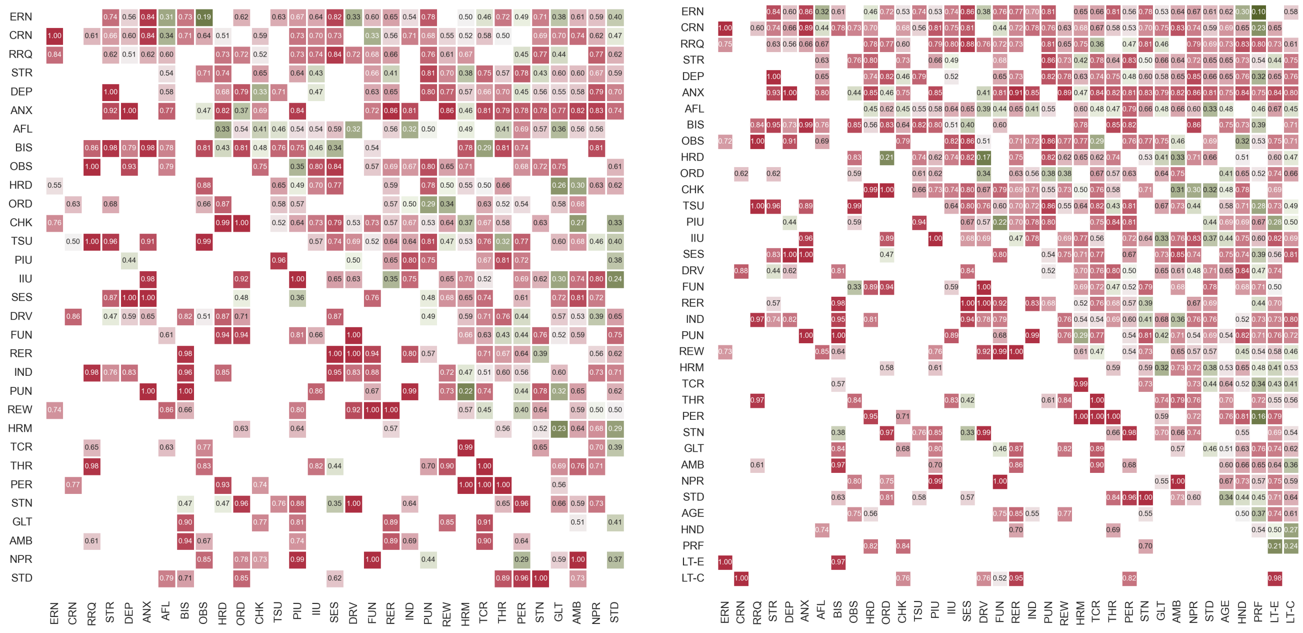

(a) The ERN network with CRN control (b) The ERN network with CRN control and covariates

Fig. 4. Results of sensitivity and specificity tests of the CRN with covariates model. The upper triangles show the specificity results; the lower triangles show the sensitivity results.

The edge-weight stability of the network models

The edge-weight stability was calculated using 1000 bootstrapped samples. Precision matrices estimated during the bootstrapping procedure can be found in *precision_matrices/bootstrap_precision_matrices* directory at `https://bit.ly/3Vn8WTk`.

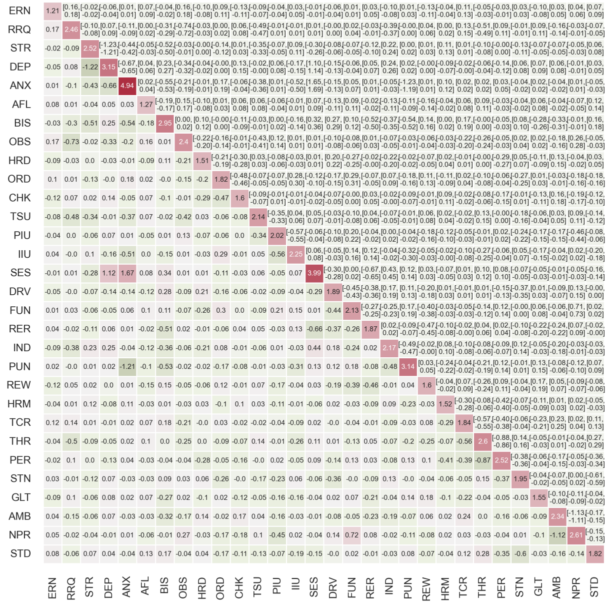

(a) The ERN network

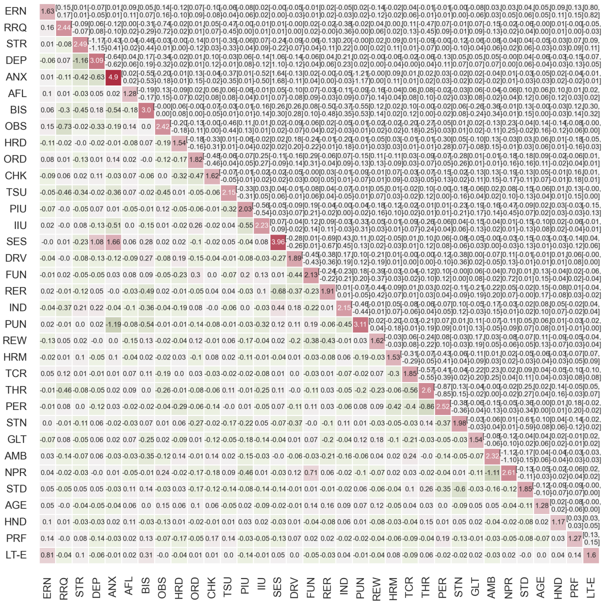

(b) The ERN network with covariates

Fig. 5. The edge-weight stability of the ERN without covariates model. Estimated edge weights (lower triangle). 95% CI interval around estimated edge weight (upper triangle).

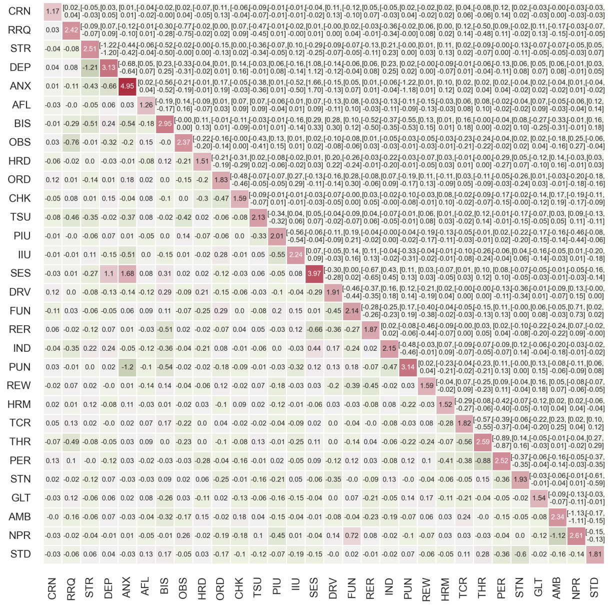

(a) The CRN network

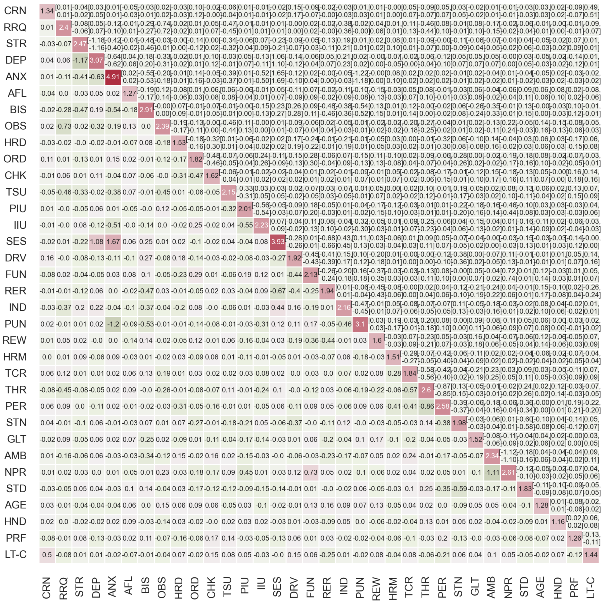

(b) The CRN network with covariates

Fig. 6. The edge-weight stability of the ERN without covariates model. Estimated edge weights (lower triangle). 95% CI interval around estimated edge weight (upper triangle).

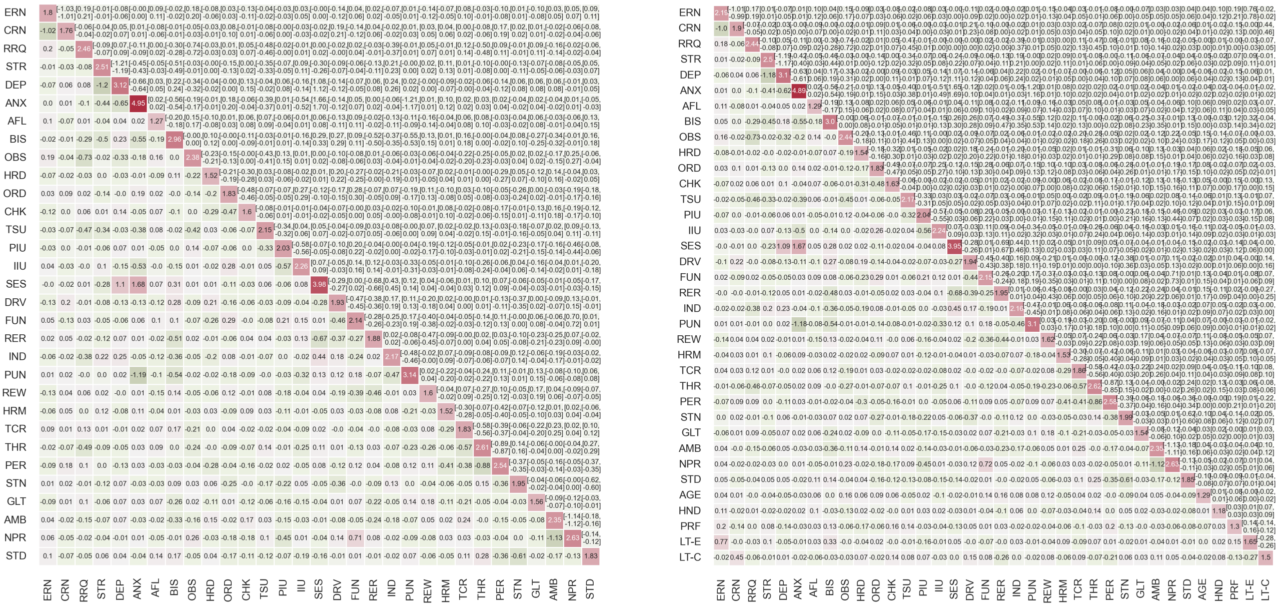

(a) The ERN network with CRN control   (b) The ERN network with CRN control and covariates

Fig. 7. The edge-weight stability of the ERN without covariates model. Estimated edge weights (lower triangle). 95% CI interval around estimated edge weight (upper triangle).

The results of the network measures stability test

The stability of a network measure is an important feature that validates the conclusions drawn from a metric value. We estimated the stability of six different network measures, including the most frequently used: betweenness, closeness, and degree centrality. We also estimated the stability of metrics less frequently used in psychology research: predictability, current flow closeness, and current flow betweenness. Similarly to the tutorial paper of Epskamp et al. [2018], betweenness and closeness, and in our case also degree centrality, did not show an adequate level of stability to draw robust conclusions. Such results suggest that centrality metrics are very sensitive to the dataset on which they are estimated, and even a dataset of similar size (95%) but with different sample content (bootstrapping is done with replacement) may yield different estimates of centrality metrics. Epskamp et al. showed that for most nodes in the network, there is no significant difference between the values of their centrality metrics. This means that the ranked order of centrality metrics in N networks estimated on bootstrapped samples might be considered random. Thus, it is not surprising that in our study, despite many nodes exhibiting statistical differences in centrality metrics' values, classic centrality metrics did not achieve a satisfactory level of stability when testing the stability using case-dropping subset bootstrap with replacement.

The high stability of the predictability metric suggests that even when the overall structure of the bootstrapped networks differs from the original network, the key neighbors, i.e., the critical connections contributing to estimating the explained variance of a node, remain present in the bootstrapped networks. Furthermore, current flow closeness, also known as information centrality, demonstrated a high level of stability, outperforming the classic closeness metric based on shortest paths. Our findings suggest that in scenarios where the overall graph structure lacks stability, which leads to unreliable estimates of shortest paths, metrics based on current flow serve as superior indicators of the most influential nodes compared to metrics based on shortest paths.

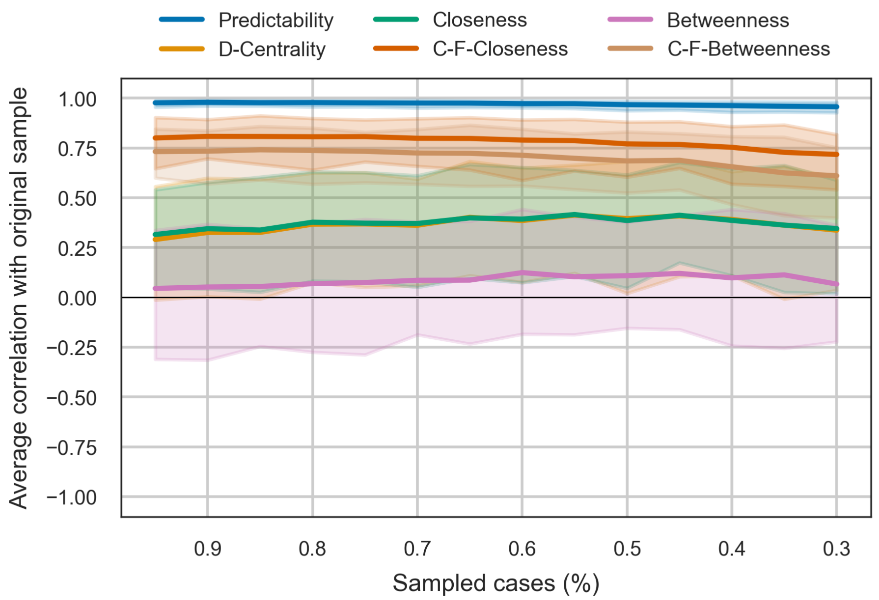

Fig. 8. Network measures stability test results. The network measures stability test followed the procedures described by Costenbader and Valente [2003]. For each percentage of sampled cases, each network measure value was calculated 100 times using bootstrapped random samples. Lines indicate the mean values, and shadows around the lines represent the range from the 2.5th percentile to the 97.5th percentile. D-Centrality = degree centrality; C-F Closeness = current flow closeness centrality; C-F Betweenness = current flow betweenness centrality.

Network with gender control

Gender is a well-documented moderator of the association between anxiety and ERN, as evidenced by studies such as Moser et al. [2016]. Although Gaussian Graphical Models require a Gaussian distribution of variables to meet their assumptions [Lauritzen, 1996], we conducted an exploratory analysis that included the gender node. Note that the results of this analysis should be interpreted with caution, as gender is a categorical variable and, in principle, should be modeled using an approach that facilitates the interaction effects of categorical variables [e.g., Haslbeck and Waldorp, 2020].

Gender was coded using -1 and 1 to represent female and male, respectively.

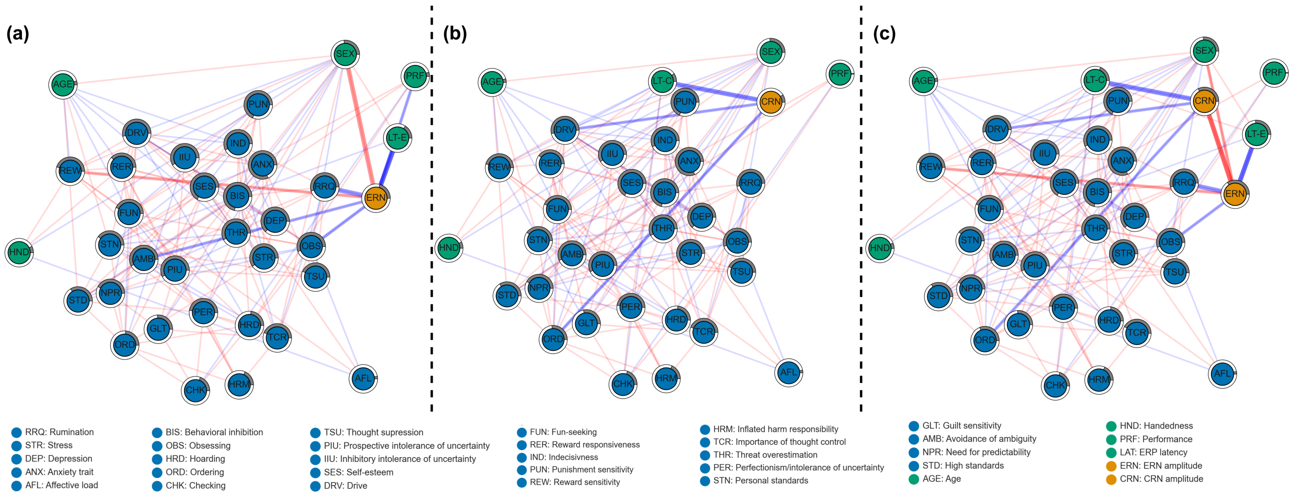

Fig. 9. ERN network with covariates (panel a), CRN network with covariates (panel b), ERN network with control for the shared CRN variance and covariates (panel c). Blue edges represent negative partial correlation associations; red edges represent positive partial correlation associations. A gray shaded area around a node represents the predictability of this node, i.e., the proportion of variance in that variable explained by the network model.

Figure source data: *gender_analysis* directory at `https://osf.io/cxkqj/`.

Significant differences in the intensity of various individual traits were observed between genders. Women exhibited higher levels of behavioral inhibition, obsessing, drive, the reward responsiveness facet of behavioral activation, perfectionism, and guilt sensitivity. Conversely, they showed lower levels of prospective intolerance of uncertainty, the fun-seeking facet of behavioral activation, reward sensitivity, threat overestimation, and shorter ERN latency. As expected, ERN amplitudes were more pronounced in women compared to men [see Moser et al., 2016; but see Riesel et al., 2019, Sandre et al., 2020, Riesel et al., 2023 for the null effect and Fischer et al., 2016, 2017, Imburgio et al., 2020 for the opposite effect]. Although several studies reported more pronounced CRN amplitudes in women [see Riesel et al., 2019], we observed no significant gender-related differences in CRN amplitudes.

When controlling for the gender effect (Figure 9, panel a), more pronounced amplitudes of ERN were associated with higher levels of rumination and obsessing, better performance, longer latencies, and lower levels of reward sensitivity. These findings were consistent with those from the network without gender control (Figure 3, panel b); adjusting for CRN amplitude did not alter these associations. Thus, in our study, gender did not moderate the relationship between ERN amplitude and individual traits. Similar to ERN results, gender was not a moderator of CRN-individual traits associations.

References

E. Costenbader and T. W. Valente. The stability of centrality measures when networks are sampled. *Social Networks*, 25(4): 283–307, Oct. 2003. ISSN 0378-8733. doi: 10.1016/S0378-8733(03)00012-1.

S. Epskamp, D. Borsboom, and E. I. Fried. Estimating psychological networks and their accuracy: A tutorial paper. *Behavior Research Methods*, 50(1):195–212, Feb. 2018. ISSN 1554-3528. doi: 10.3758/s13428-017-0862-1.

A. G. Fischer, C. Danielmeier, A. Villringer, T. A. Klein, and M. Ullsperger. Gender Influences on Brain Responses to Errors and Post-Error Adjustments. *Scientific Reports*, 6(1):24435, Apr. 2016. ISSN 2045-2322. doi: 10.1038/srep24435.

A. G. Fischer, T. A. Klein, and M. Ullsperger. Comparing the error-related negativity across groups: The impact of error- and trial-number differences. *Psychophysiology*, 54(7):998–1009, 2017. ISSN 1469-8986. doi: 10.1111/psyp.12863. _eprint: https://onlinelibrary.wiley.com/doi/pdf/10.1111/psyp.12863.

J. M. B. Haslbeck and L. J. Waldorp. mgm: Estimating Time-Varying Mixed Graphical Models in High-Dimensional Data. *Journal of Statistical Software*, 93:1–46, Apr. 2020. ISSN 1548-7660. doi: 10.18637/jss.v093.i08.

M. J. Imburgio, I. Banica, K. E. Hill, A. Weinberg, D. Foti, and A. MacNamara. Establishing norms for error-related brain activity during the arrow Flanker task among young adults. *NeuroImage*, 213:116694, June 2020. ISSN 1053-8119. doi: 10.1016/j.neuroimage.2020.116694.

S. L. Lauritzen. *Graphical Models*. Oxford Statistical Science Series. Oxford University Press, Oxford, New York, May 1996. ISBN 978-0-19-852219-5.

J. S. Moser, T. P. Moran, C. Kneip, H. S. Schroder, and M. J. Larson. Sex moderates the association between symptoms of anxiety, but not obsessive compulsive disorder, and error-monitoring brain activity: A meta-analytic review. *Psychophysiology*, 53(1): 21–29, 2016. ISSN 1469-8986. doi: 10.1111/psyp.12509. _eprint: https://onlinelibrary.wiley.com/doi/pdf/10.1111/psyp.12509.

A. Riesel, J. Klawohn, R. Grützmann, C. Kaufmann, S. Heinzel, K. Bey, L. Lennertz, M. Wagner, and N. Kathmann. Error-related brain activity as a transdiagnostic endophenotype for obsessive-compulsive disorder, anxiety and substance use disorder. *Psychological Medicine*, 49(7):1207–1217, May 2019. ISSN 0033-2917, 1469-8978. doi: 10.1017/S0033291719000199.

A. Riesel, K. Härpfer, L. Thoma, N. Kathmann, and J. Klawohn. Associations of neural error-processing with symptoms and traits in a dimensional sample recruited across the obsessive-compulsive spectrum. *Psychophysiology*, 60(2):e14164, Feb. 2023. ISSN 1469-8986. doi: 10.1111/psyp.14164.

A. Sandre, I. Banica, A. Riesel, J. Flake, J. Klawohn, and A. Weinberg. Comparing the effects of different methodological decisions on the error-related negativity and its association with behaviour and gender. *International Journal of Psychophysiology*, 156: 18–39, Oct. 2020. ISSN 0167-8760. doi: 10.1016/j.ijpsycho.2020.06.016.
